# Single-cell and machine learning integration reveals OS-driven CCND1 promotes an aggressive phenotype in papillary thyroid carcinoma

**DOI:** 10.3389/fimmu.2025.1722524

**Published:** 2026-01-14

**Authors:** Jiaxi Wang, Qingyi Zhu, Jingyi Bie, Yueyu Han, Hanqing Liu, Chuang Chen

**Affiliations:** 1 Department of Breast and Thyroid Surgery, Renmin Hospital of Wuhan University, Wuhan, China; 2 Department of Cardiovascular Surgery, Renmin Hospital of Wuhan University, Wuhan, China; 3 College of Medicine, Yan’an University, Yan’an, China; 4 Department of Thyroid Surgery, The First Affiliated Hospital, School of Medicine, Zhejiang University, Hangzhou, China

**Keywords:** CCND1, oxidative stress, papillary thyroid carcinoma, single-cell RNA sequencing, Sox4, TFF3

## Abstract

**Background:**

Papillary thyroid carcinoma (PTC) is the most common thyroid malignancy, with rising incidence worldwide. Oxidative stress (OS), characterized by an imbalance between reactive oxygen species (ROS) and antioxidant defenses, plays a critical role in tumor initiation and progression. However, the specific relationship between OS and PTC remains underexplored, highlighting the need for further investigation. This study aims to identify OS-related biomarkers in PTC that could potentially be used for clinical diagnosis and treatment.

**Methods:**

Single-cell RNA sequencing data from PTC and normal thyroid tissues were analyzed using multiple gene set scoring and differential expression methods to evaluate OS levels across different cell types. Integrated bioinformatics analysis, including WGCNA and machine learning models, was employed to select candidate biomarkers, which were then validated in independent datasets. Pseudotime analysis and CellChat were conducted to explore cell dynamics within the tumor microenvironment. An oxidative stress model was established in TPC-1 cells using hydrogen peroxide treatment. The levels of OS and changes in tumor cell proliferative capacity were assessed through western blotting, immunoblotting, ROS detection, and cell viability assays.

**Results:**

The study revealed that *CCND1* and *SOX4* were highly expressed in PTC, promoting tumor cell proliferation, invasion, and maintaining an undifferentiated state. Both genes were closely linked to OS, which amplified their expression and enhanced tumor growth and immune evasion. *CCND1* was particularly involved in M2 macrophage polarization via the PROS1-AXL pathway, while *SOX4* regulated angiogenesis through the MDK pathway. In contrast, *TFF3* expression was significantly lower in PTC, suggesting a tumor-suppressive role, potentially through modulating immune responses and reducing OS.

**Conclusion:**

*CCND1* is identified as a key oncogene in PTC, whose high expression promotes tumor progression through OS-related pathways like PI3K/AKT and MAPK. Our *in vitro* findings specifically validate that OS directly drives *CCND1* overexpression and subsequent cell proliferation. Conversely, *SOX4* also acts as an oncogene, and *TFF3* as a potential tumor suppressor, both linked to OS. Targeting *CCND1* and its OS-mediated regulatory pathways offers a promising therapeutic strategy for PTC.

*CCND1*, oxidative stress, papillary thyroid carcinoma, single-cell RNA sequencing, *SOX4*, *TFF3*.

## Introduction

Thyroid cancer is a common malignancy, and it is also the most common malignant tumor of the endocrine system globally (1). Papillary thyroid carcinoma (PTC) is the most prevalent form of thyroid cancer, accounting for approximately 90% of all thyroid malignancies (2, 3). Its incidence has been rising significantly, particularly among women, with studies indicating an increase of over 200% in certain populations over the past few decades (2, 4). Despite its generally favorable prognosis, PTC presents considerable health burdens due to potential recurrence and the psychological impact of a cancer diagnosis on patients (5). Although rare, PTC can metastasize distant sites like the lungs and bones, and are often associated with a poor prognosis (6). When PTC patients undergo radioactive iodine therapy, the presence of specific molecular markers may lead to treatment resistance, which is closely linked to the patient’s survival rate and recurrence risk (7).

Oxidative stress (OS) has emerged as a critical factor in cancer biology, influencing tumor initiation, progression, and metastasis (8). OS is characterized by an imbalance between reactive oxygen species (ROS) production and the body’s antioxidant defenses, leading to cellular damage (9). Elevated ROS levels can cause DNA damage, genomic instability, and the activation of pro-tumorigenic signaling pathways, thereby contributing to tumorigenesis (10). In thyroid cancer, increased OS has been associated with more aggressive tumor features (11).

The mechanisms by which OS influences cancer progression are multifaceted. ROS can activate various signaling pathways, including the mitogen-activated protein kinase (MAPK) and phosphatidylinositol-3-kinase (PI3K) pathways, which are known to promote cell proliferation and survival (12). Furthermore, chronic inflammation, often present in thyroid cancer, is a significant source of ROS, creating a vicious cycle that exacerbates OS and enhances tumorigenesis (13).

Research indicates that the OS status in thyroid tumors is significantly higher than that in normal tissues, highlighting its potential role as a new risk factor for thyroid cancer (11). Wang et al. reported that patients with thyroid cancer exhibited elevated total oxidant status and OS indices, further establishing the link between OS and thyroid malignancies (14). The clinical implications of this association are profound, as OS not only contributes to thyroid cancer development but may also increase the risk of cardiovascular diseases and other malignancies associated with oxidative damage (15).

Despite the established role of OS in various cancers, the specific relationship between PTC and OS remains underexplored. This gap in the literature underscores the necessity for further research to elucidate the molecular mechanisms linking OS to PTC, which may lead to novel therapeutic strategies targeting OS pathways. Understanding the interplay between OS and PTC can provide insights into the disease’s pathophysiology and potentially improve patient outcomes through targeted interventions.

## Materials and methods

### Single-cell RNA sequencing data collection and preprocessing

The single-cell RNA sequencing (scRNA-seq) data were obtained from the Gene Expression Omnibus (GEO) database (https://www.ncbi.nlm.nih.gov/geo/) under accession number GSE184362. The dataset includes samples from seven patients with papillary thyroid carcinoma (PTC) and six healthy controls (CON). The raw count matrix was processed using the Seurat V5 package in R (16). Cells expressing fewer than 300 detected genes or with mitochondrial content exceeding 10% were filtered out to remove low-quality cells. Following this quality control step, data normalization was calculated using Seurat’s log normalization method, and highly variable genes were identified for downstream analysis.

### Clustering and UMAP visualization

Following normalization, batch effect correction was calculated using the Harmony integration method within the Seurat package, followed by principal component analysis (PCA) for dimensionality reduction. The first 10 principal components (PCs) were employed to identify clusters based on a shared nearest neighbor (SNN) graph, with the resolution parameter optimized to distinguish different cell populations. Cells were divided into 28 clusters based on similar gene expression profiles, and these clusters were visualized in two dimensions using Uniform Manifold Approximation and Projection (UMAP).

### Cell type annotation and marker gene analysis

Cell types within the identified clusters were annotated using a combination of known marker genes and differential gene expression analysis. The distribution of cells between the PTC and CON groups was compared to assess differences in cell populations under varying conditions. Marker genes corresponding to cell types were referenced from the CellMarker database (http://xteam.xbio.top/CellMarker/) and the BioLegend database (https://www.biolegend.com/en-us/phenotyping/cell-markers), and previous research reports. For each cluster, the Seurat `FindMarkers` function was used to identify the top markers and key cell types. Violin plots and dot plots were generated to visualize the expression of marker genes across various cell types. Density plots were used to further confirm the specificity of marker expression, ensuring that the marker genes were predominantly expressed in the expected cell types.

### OS scoring and analysis

To assess OS levels in PTC and CON samples, a gene set of 807 OS-related genes was obtained from the GeneCards database (17). Five gene set enrichment scoring methods—AddModuleScore, Aucell, UCell, singscore, and ssGSEA—were applied to compute OS-related scores for each cell (16, 18–21). The scores from each method were normalized, and a composite OS score was derived by integrating the results from all five methods. These scores were used to compare OS levels across different cell types and to perform statistical comparisons between the PTC and CON groups. Cells were categorized into three groups based on their composite OS scores: Low (below the 25th percentile), High (above the 75th percentile), and Median (between the 25th and 75th percentiles). Finally, the OS results were visualized using violin plots, dot plots, density plots, and UMAP projections.

### Differentially expressed genes analysis based on OS levels

Differential expression analysis was calculated between high OS and low OS cells using the `FindMarkers` function (**Supplementary Table 1**) (16). This analysis focused on identifying genes that were upregulated in the high OS group. The significant OS-related genes were visualized using a volcano plot, highlighting the genes with the most pronounced differential expression.

### Co-expression network construction and analysis based on hdWGCNA

High-dimensional Weighted Gene Co-expression Network Analysis (hdWGCNA) was employed to identify gene modules associated with PTC. A co-expression network was constructed using the gene expression matrix from the scRNA-seq data. The soft threshold was selected based on scale-free topology fitting, mean connectivity, and median connectivity, with a soft threshold (*β*) of 6 chosen to ensure a robust network structure. Gene modules were identified through hierarchical clustering, and each module was assigned a unique color. Module eigengenes (MEs) were calculated, and the module membership (MM) values were used to identify key genes within each module (**Supplementary Table 2**). Dot plots and network graphs were generated to illustrate the relationships between modules and cell types, as well as the connections among key genes. A heatmap was also generated to show the correlations between different modules, highlighting their interactions and potential functional relationships.

### Functional enrichment analysis of OS-related genes

Genes related to PTC and OS were identified by intersecting the genes from hdWGCNA with the upregulated DEGs from the high/low OS group comparison. A Venn diagram was used to visualize the overlap between the genes identified by hdWGCNA and the upregulated DEGs in the high OS group (**Supplementary Table 3**). A heatmap was generated to display the expression of these genes between the PTC and CON groups. The intersected genes were subjected to Gene Ontology (GO) and Kyoto Encyclopedia of Genes and Genomes (KEGG) pathway enrichment analysis using the clusterProfiler package (22–24). GO terms related to biological processes (BP), cellular components (CC), and molecular functions (MF) were identified. Enriched pathways were visualized using bar plots, highlighting key functional and pathway-level associations with OS and PTC.

### Machine learning for hub gene identification

To optimize the selection of hub OS-related genes, bulk RNA-seq data from GSE33630 (49 PTC and 45 CON samples) and GSE60542 (33 PTC and 30 CON samples), totaling 82 PTC and 75 CON samples, were processed after batch effect removal using the sva package, and missing values were imputed using the k-Nearest Neighbors (KNN) algorithm (25). Five machine learning algorithms were applied: LASSO regression, support vector machine (SVM) with recursive feature elimination (RFE), random forest, Boruta, and XGBoost (26–30). LASSO regression was used to shrink the coefficients of irrelevant genes to zero, selecting the optimal λ value to minimize error. XGBoost was utilized to rank gene importance. A Venn diagram was generated to identify genes shared among the different machine learning models.

### Validation of hub genes in independent datasets

The expression patterns of *CCND1*, *SOX4*, and *TFF3* were validated in independent scRNA-seq and bulk RNA-seq datasets. Violin plots were used to compare the expression levels of these genes between PTC and CON samples. Dot plots were employed to examine the cell type-specific expression of these genes, while UMAP projections visualized their spatial distribution across cell populations. Additionally, the expression of the hub genes was explored in metastatic and non-metastatic tumors using PTC samples and normal controls from the TCGA database. GEPIA2 was used to analyze survival data, investigating the clinical relevance of *CCND1*, *SOX4*, and *TFF3*. Kaplan-Meier survival curves were generated to assess the association between gene expression and disease-free survival (DFS).

### Pseudotime and cell chat analysis

CytoTRACE was used to assess the differentiation potential of cells based on gene expression profiles (31). Pseudotime analysis was calculated using the Monocle package to investigate the differentiation dynamics of *CCND1*+/-, *SOX4*+/-, and *TFF3*+/- Thyroid cells (32). Pseudotime trajectories were visualized to show the progression of *CCND1*+/-, *SOX4*+/-, and *TFF3*+/- Thyroid cells along differentiation paths. Cell Chat analysis was conducted using the CellChat package to identify ligand-receptor interactions and quantify the communication probability across different cell types. Network graphs, dot plots, and heatmaps were generated to illustrate the key signaling pathways, highlighting the roles of *CCND1*, *SOX4*, and *TFF3* in regulating tumor microenvironment interactions. This analysis emphasized their involvement in shaping the communication landscape between tumor and surrounding cells.

### Protein-protein interaction network and correlation analysis

The protein-protein interaction (PPI) network for hub genes, including *CCND1*, *SOX4*, and *TFF3*, was constructed using the STRING database, with a minimum interaction score threshold of 0.4 to ensure high-confidence interactions (33). Through network visualization, *CCND1* was identified as a central regulatory gene, revealing its complex interactions with *TP53*, *SOX4*, *TFF3*, and other genes. Pearson correlation analysis was calculated using TCGA data to calculate the correlation coefficients (R) and p-values between *CCND1*, *SOX4*, *TFF3*, and *TP53*. A p-value of less than 0.05 was considered statistically significant. This analysis uncovered the potential co-regulatory roles of these genes in PTC, providing insights into their collective influence on tumor development and progression.

### Cell culture and transfection

The normal human thyroid cell line Nthy-ori3–1 and two human papillary thyroid carcinoma cell lines, TPC-1, were obtained from Keycell Biotechnology Co., Ltd. (Wuhan, China). Cells were maintained in RPMI 1640 medium containing 10% serum and 1% penicillin-streptomycin. All cell lines were cultured at 37°C in a humidified atmosphere of 5% CO_2_;.

For transfection, cells were transfected with either specific CCND1-targeting si-RNA sequences or a scrambled non-targeting siRNA control (referred to as Si-Control) (Sangon Biotech, Shanghai, China) using Lipofectamine 3000 Reagent (Thermo Fisher Scientific Inc.), according to the manufacturer’s instructions. Transfection efficiency was verified by Western blot analysis. To induce oxidative stress, hydrogen peroxide (H_2_;O_2_;; CAS: 7722-84-1, Sigma-Aldrich) was applied to the cells. For experimental grouping, samples were designated as follows: 1) NC-Si-Control (cells transfected with scrambled siRNA without H_2_;O_2_; treatment); 2) OS-Si-Control (cells transfected with scrambled siRNA and treated with H_2_;O_2_;); 3) NC-Si-CCND1 (cells transfected with CCND1-siRNA without H_2_;O_2_; treatment); and 4) OS-Si-CCND1 (cells transfected with CCND1-siRNA and treated with H_2_;O_2_;). Prior to these treatments, cells were serum-starved for 12 hours using RPMI 1640 medium supplemented with 1% serum.

### Western blotting

Cells were washed with PBS and lysed on ice using RIPA buffer (Beyotime, #P0013K, China) supplemented with a protease inhibitor cocktail (Servicebio, #G2006-250UL, China) and phenylmethylsulfonyl fluoride (Servicebio, #G2008-1ML, China). The lysates were subjected to ultrasonication followed by centrifugation at 10,000 × g for 12 min at 4°C to remove cellular debris. Equal amounts of protein were separated by 8–10% SDS-PAGE and electrophoretically transferred onto polyvinylidene difluoride (PVDF) membranes (Merck Millipore, #ISEQ00010, USA). The membranes were blocked with 5% skim milk in PBST for 1.5 h at room temperature and then incubated overnight at 4°C with primary antibodies against Cyclin D1 (1:1000; Abways, #CY5404, China) and β-actin (1:1000; Proteintech, #66009-1-Ig, USA). After washing, the membranes were probed with HRP-conjugated Goat Anti-Mouse IgG (1:7000; Proteintech, #SA00001-1, USA) or Goat Anti-Rabbit IgG (1:7000; Proteintech, #SA00001-2, USA) secondary antibodies. Protein bands were visualized using a chemiluminescence imaging system (BIO-RAD, USA), and band intensities were quantified with accompanying software. A minimum of three independent experiments were conducted.

### Cell viability

Cells were seeded in 96-well plates at a density of 1×10_5_ cells/mL (100 µL per well) and cultured under standard conditions. At 12, 24, and 48 h post-seeding, cell viability was assessed using a Cell Counting Kit-8 (Beyotime, #C0037, China). Absorbance was measured at 570 nm using a microplate reader.

### Immunohistochemistry

Paraffin-embedded sections were deparaffinized and rehydrated following heating at 60°C. Antigen retrieval was conducted via microwave irradiation. Endogenous peroxidase activity was inhibited with hydrogen peroxide. Sections were blocked with immunostaining blocking buffer (Beyotime, #P0102, China) at 37°C. They were then incubated with a primary antibody against Cyclin D1 (1:200; Abways, #CY5404, China) at 4°C overnight. After washing, sections were treated with HRP-conjugated Goat Anti-Rabbit IgG (H+L) (1:7,000; Proteintech, #SA00001-2, USA). Detection was performed using a DAB Horseradish Peroxidase Color Development Kit (Beyotime, #P0203, China). Counterstaining was carried out with hematoxylin (Beyotime, #C0107, China), followed by differentiation in acid alcohol (Beyotime, #C0163S, China) and thorough rinsing. Finally, sections were dehydrated, cleared in xylene, and mounted. Images were acquired using an Olympus BX53 microscope (Olympus Corporation, Japan).

### Statistical analysis

All data are expressed as the mean ± SD of at least three separate experiments. Statistical significance was evaluated with a two-tailed paired t test by using GraphPad Prism 7.0 software. A *p* value of <0.05 was considered to indicate significance.

## Results

### The single-cell landscape of PTC reveals immune remodeling and the expansion of thyroid and myeloid lineages

**Figure 1:**
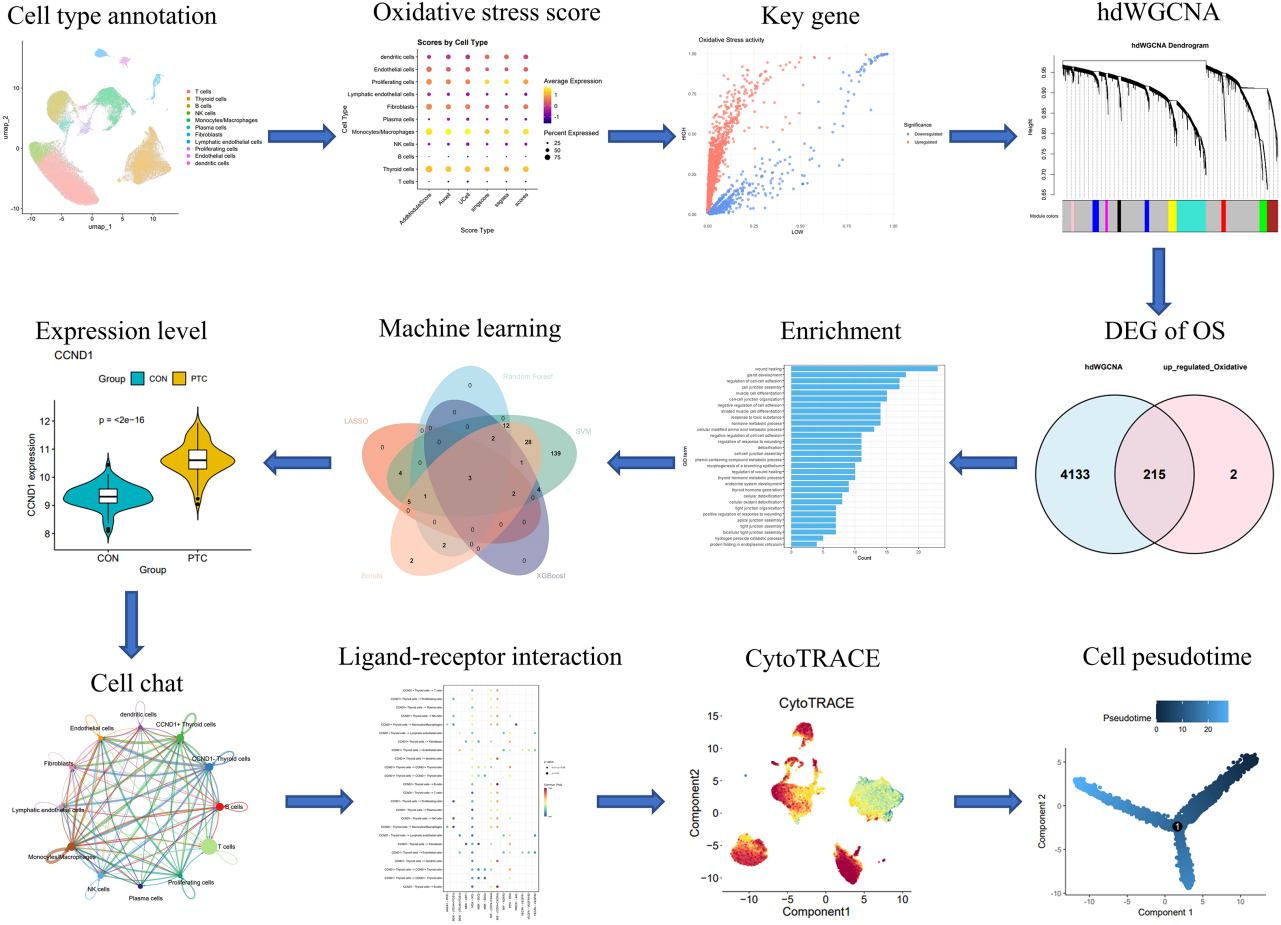
The workflow diagram of this study.

**Figure 2:**
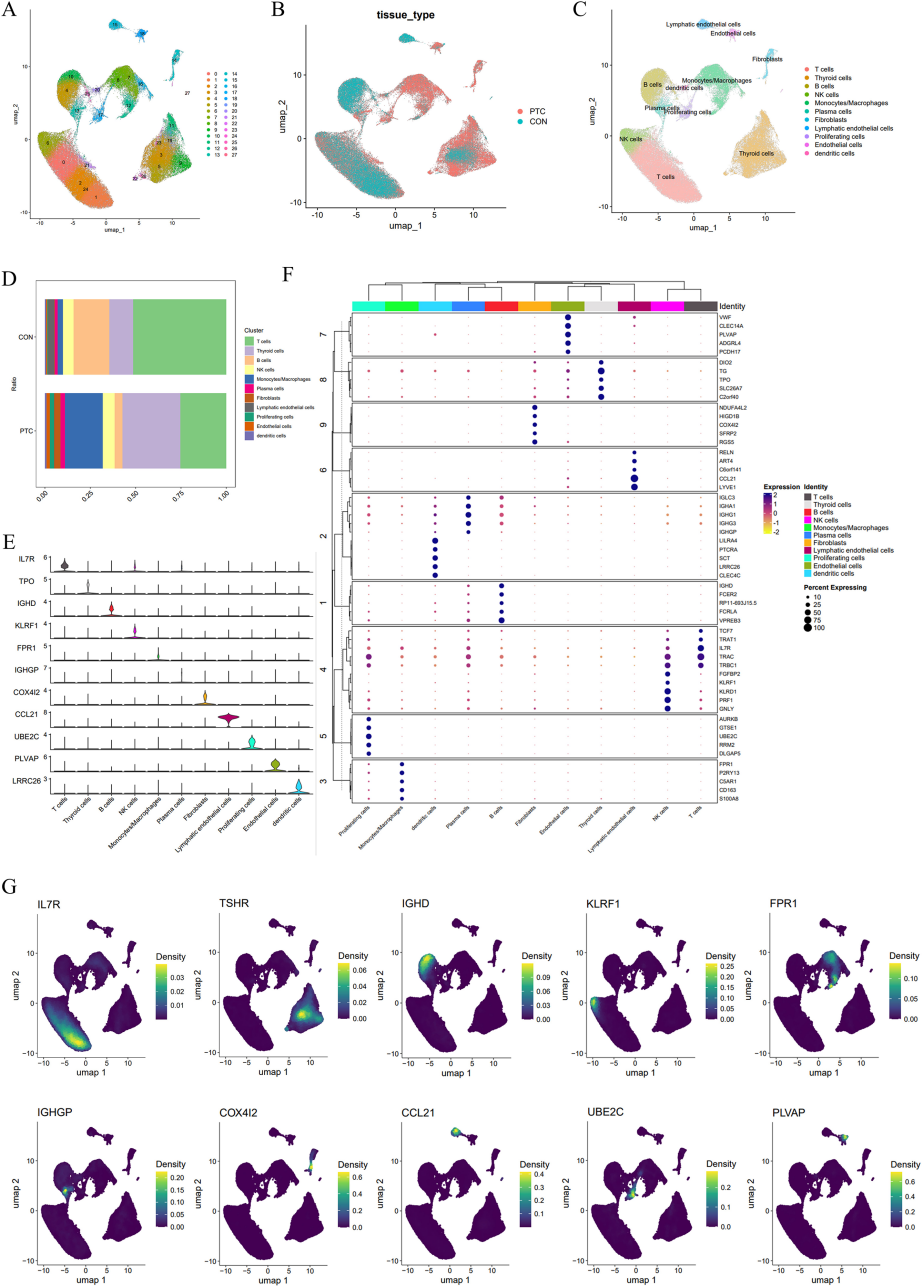
Single-cell RNA sequencing analysis reveals distinct cellular populations and gene expression profiles in PTC and normal thyroid tissue. **(A)** UMAP plot showing the clustering of cells based on their gene expression profiles. Each cluster represents distinct cell populations identified across both PTC and CON samples. **(B)** UMAP plot colored by tissue type, comparing cells from PTC and CON samples. **(C)** UMAP plot highlighting the different cell types, including T cells, macrophages/monocytes, and thyroid cells, among others, in both PTC and normal tissues. **(D)** Scale plot showing the distribution of cell types between PTC and normal thyroid tissues, with PTC exhibiting an increased proportion of certain cell populations. **(E)** Violin plots showing the expression levels of selected marker genes, such as IL7R, TPO, and IGHD, across various cell types. **(F)** Dot plot illustrating the expression of key marker genes across different cell types identified in both PTC and normal thyroid tissues. The size and color intensity of the dots indicate the percentage of cells expressing each marker and the average expression level, respectively. **(G)** UMAP density plots demonstrating the distribution of cell markers, including IL7R, TSHR, and CCL21, across the UMAP space. These genes show distinct expression patterns in various cell types.

The workflow diagram of this study was in **Figure 1**. We used Seurat to analyze scRNA-seq data from seven PTC patients and six CON patients obtained from GEO184362, identifying 28 clusters. Cells within each cluster exhibited similar gene expression profiles, and we visualized these clusters using UMAP. On the UMAP plot, adjacent clusters may represent the same cell type or closely related cell types (**Figure 2A**). The distribution of cells from PTC and CON groups differed, with some cell populations dominated by CON cells, while others were primarily composed of PTC cells. Notably, thyroid cells and monocytes/macrophages were more abundant in the PTC group, while B cells and T cells were more prevalent in the CON group (**Figures 2B–D**).

Different cell types expressed distinct markers, and we used violin plots and clustered dot plots to display the top-ranked genes for each cell type. T cells highly expressed IL7R, and thyroid cells had high expression of TPO, both well-known characteristic markers for these cell types. While NK cells and T cells shared some markers, NK cells also expressed unique markers, consistent with established biological knowledge (**Figures 2E, F**). The expression of these cell type-specific markers was presented in density plots, showing high expression in the corresponding cell types and minimal expression in other types (**Figure 2G**). The cell types corresponding to different markers in **Figure 2G** are as follows: T cell: IL7R; Thyroid cells: TSHR; B cells: IGHD; NK cells: KLRF1; Monocytes/macrophages: FPR1; Plasma cells: IGHGP; Fibroblasts: COX4I2; Lymphatic endothelial cells: CCL21; Proliferating cells: UBE2C; Endothelial cells: PLVAP.

### OS is predominantly enriched in thyroid cells and monocytes/macrophages within the PTC microenvironment

**Figure 3:**
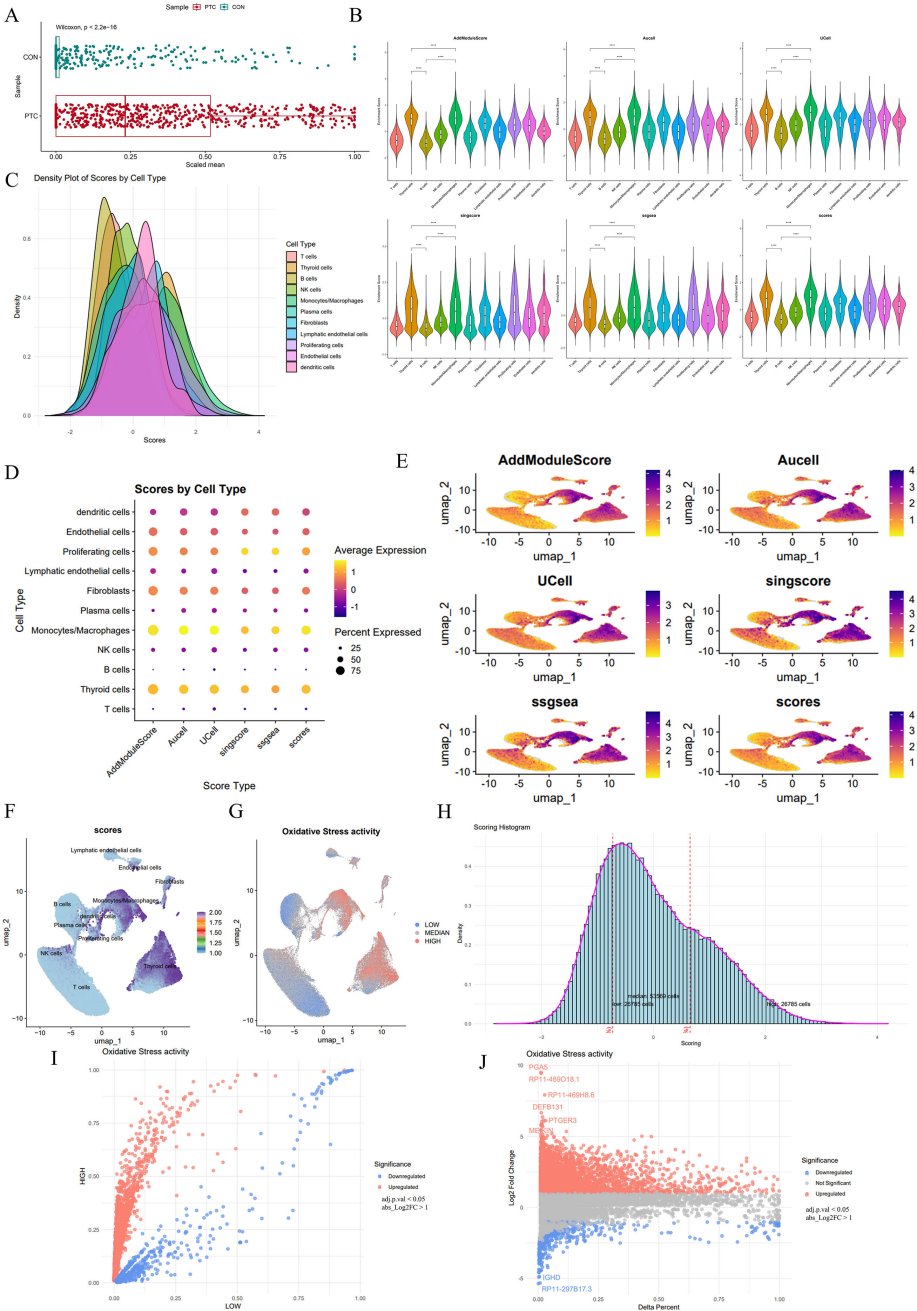
OS scoring reveals elevated OS activity in specific cell types within PTC. **(A)** Scatter plot comparing OS scores between PTC and CON (*p* < 0.01). **(B)** Violin plots of OS scores across cell types, using five different scoring methods: AddModuleScore, Aucell, UCell, singscore, ssGSEA and composite OS score. ****p < 0.0001 **(C)** Density plot of composite OS scores across different cell types. **(D)** Dot plot illustrating OS scores across cell types based on the five scoring methods. **(E)** UMAP plots showing the distribution of OS scores across the five methods and composite score. **(F)** UMAP plot of the composite OS score distribution across cell types. **(G)** UMAP plot categorizing cells into high, median, and low OS activity groups based on score thresholds. **(H)** Histogram depicting the distribution of OS scores across all cells. The 75th percentile and 25th percentile thresholds define high, median, and low OS groups. **(I)** Scatter plot showing the differential gene expression between high and low OS groups. **(J)** Volcano plot displaying the fold change and significance of genes differentially expressed between high and low OS groups.

To explore the role of OS in PTC, we scored PTC and CON samples using an OS-related gene set from GeneCards. We calculated the total expression of all genes in the OS-related gene set and calculated statistical tests, showing that the expression of OS-related genes in the PTC group was significantly higher than in the CON group (*p* < 0.01) (**Figure 3A**). Five gene set scoring methods were employed: AddModuleScore, Aucell, UCell, singscore, and ssGSEA, to evaluate OS levels in each cell type. After normalizing the results of the five methods, a composite score was generated.

Across all five methods, monocytes/macrophages and thyroid cells consistently showed the highest OS scores, while B cells, T cells, and other immune cells generally scored lower. After normalizing the results of the five methods, a composite score was generated. To minimize errors from different methods, the five OS-related scores were normalized and combined into a single composite score. Under this system, monocytes/macrophages remained the highest-scoring cell type, followed by thyroid cells and proliferating cells, while other cell types had lower OS-related scores (**Figure 3B**). A density plot was used to display the OS-related scores and their distribution across different cell types. Thyroid cells had the highest peak score and a higher density than monocytes/macrophages. Monocytes/macrophages had the largest extreme value (**Figure 3C**).

The results of the five scoring methods, along with the composite score, were visualized in a dot plot, showing that monocytes/macrophages and thyroid cells had the highest scores. Notably, proliferating cells also had a relatively high composite score, despite their lower proportion of expression (**Figure 3D**). These findings support the idea that OS plays a central role in specific cell populations within the PTC tumor microenvironment.

The UMAP plot of predicted OS activity revealed distinct high-OS cell clusters, predominantly in thyroid and Monocytes/Macrophages populations. Other cell types, such as fibroblasts and endothelial cells, also showed moderate OS activity, but to a lesser extent (**Figure 3E**). When plotting OS scores across cell types based on the composite score, thyroid cells formed the most concentrated high OS cluster, while lymphatic endothelial cells showed lower activity (**Figure 3F**).

A histogram of OS score distribution across all cells showed that cells scoring above the 75th percentile were classified into the high OS group, those below the 25th percentile into the low OS group, and those between the 25th and 75th percentiles into the median OS group (**Figure 3H**). Mapping the OS groups onto the UMAP plot revealed that high OS cells were predominantly clustered in thyroid cells and monocytes/macrophages (**Figure 3G**). Differential gene expression analysis between high and low OS groups identified a set of upregulated genes associated with high OS, such as PGA5 and DEFB131 (**Figures 3I, J**).

### Cell-type specific gene co-expression networks characterize the heterogeneous transcriptomic architecture of PTC

**Figure 4:**
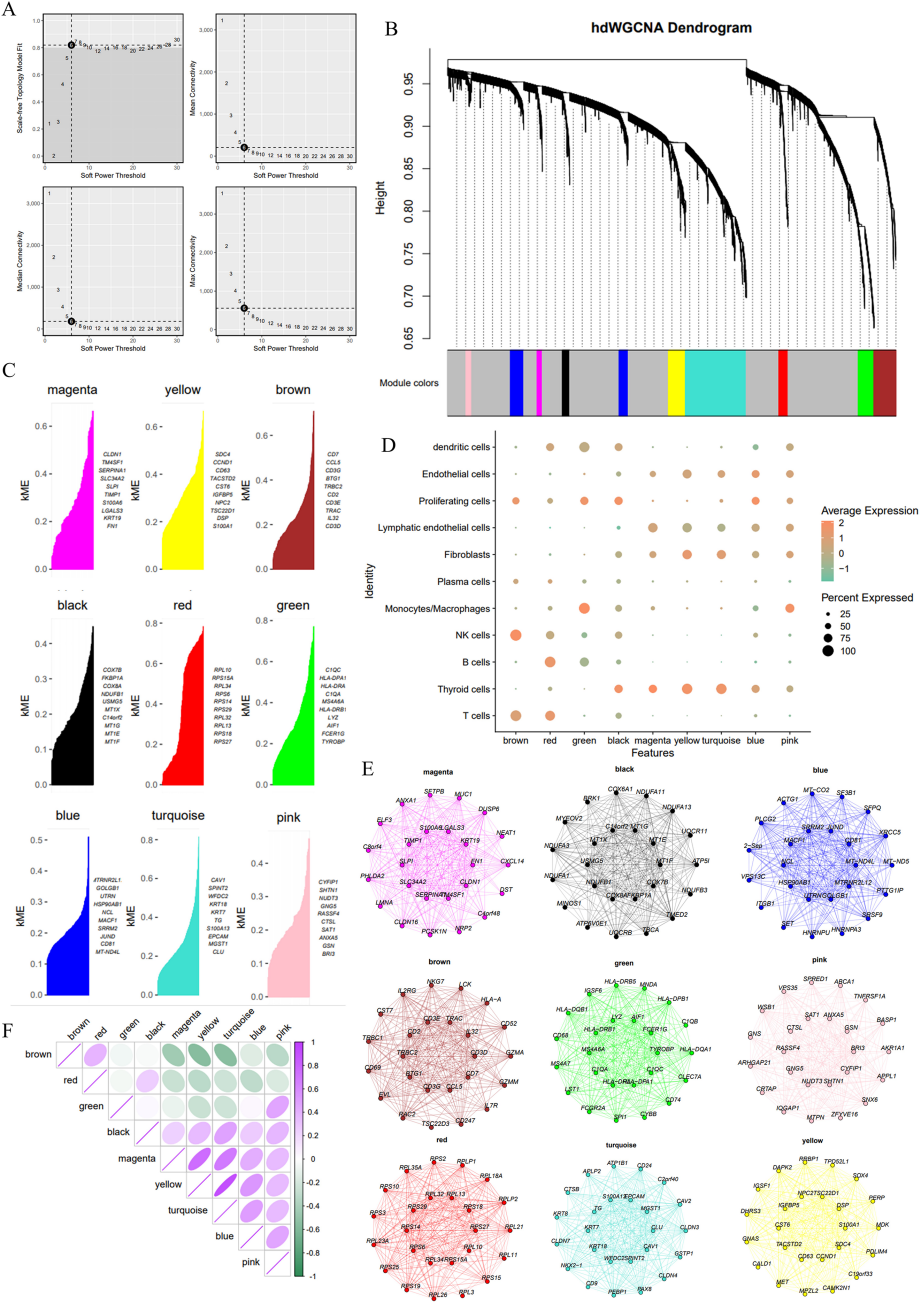
Co-expression network analysis identifies gene modules associated with PTC using hdWGCNA. **(A)** Soft threshold selection plots based on scale-free topology fit, mean connectivity, and median connectivity, with a threshold of 6 chosen for constructing the network. **(B)** Hierarchical clustering dendrogram of genes grouped into distinct modules, each represented by a unique color. **(C)** KME (module eigengene) plots for selected modules, showing the hub genes with the highest KME values for each module. **(D)** Dot plot illustrating the expression of module genes across different cell types. The size of the dots represents the percentage of cells expressing genes in each module, and the color intensity corresponds to average expression levels. **(E)** Network graphs for selected modules. **(F)** Heatmap of correlations between modules, showing positive and negative correlations between gene modules, indicating potential functional overlap or distinct roles within the PTC microenvironment.

We constructed and analyzed a co-expression network using hdWGCNA to identify gene modules associated with PTC. We assessed the soft threshold based on scale-free topology fitting, mean connectivity, and median connectivity. A soft threshold of 6 was chosen to ensure a robust co-expression network (**Figure 4A**). The hierarchical clustering dendrogram illustrates how genes were divided into distinct modules, with each module represented by a unique color. Several gene modules were identified, including magenta, yellow, brown, black, red, green, blue, turquoise, and pink, each containing distinct co-expressed genes (**Figure 4B**). The expression of module genes varied across different cell groups (**Supplementary Figure 1A**).

To further characterize these modules, KME (module eigengene) values were plotted for each module, highlighting the most important key genes (**Figure 4C**). For instance, in the magenta module, genes such as *CLDN1* and *TM4SF1* exhibited the highest KME values, indicating their central role in the co-expression network. Similarly, key genes in the yellow, brown, black, red, and green modules, including *CDC6*, *CD7*, and *COX7B*, may play crucial roles in the OS response within PTC. A dot plot displayed the relationships between these gene modules and different cell types. Each module was associated with specific cell populations. For example, the brown and red modules were highly expressed in T cells, whereas the magenta and blue modules were more highly expressed in thyroid cells. This suggests that these gene networks may be cell type-specific, with certain modules being more relevant to specific cell populations within the PTC microenvironment (**Figure 4D**).

To visualize the intricate relationships between key genes within each module, network graphs were generated for selected modules. These graphs revealed densely co-expressed networks with strong connectivity between the central genes within each module (**Figure 4E**). A heatmap assessed the relationships between the identified modules. Positive correlations were observed between several modules, such as brown and red, black and magenta, suggesting potential functional overlap or shared regulatory pathways between these gene networks. In contrast, some modules, such as brown and yellow, showed negative correlations, indicating differing roles in the PTC tumor environment (**Figure 4F**).

A UMAP plot was used to display the relationships between the modules and representative genes, showing that the green module appeared more isolated, while the red and brown modules clustered closely together, with other modules forming a main cluster (**Supplementary Figure 1B**). Overall, the detailed analysis of the co-expression network highlighted several key gene modules, each associated with distinct cell populations, potentially contributing to different biological processes within PTC. These findings lay the groundwork for further research into the functional roles of these modules and their potential as therapeutic targets.

### OS-associated gene signatures drive metabolic reprogramming, tissue repair, and extracellular matrix remodeling

**Figure 5:**
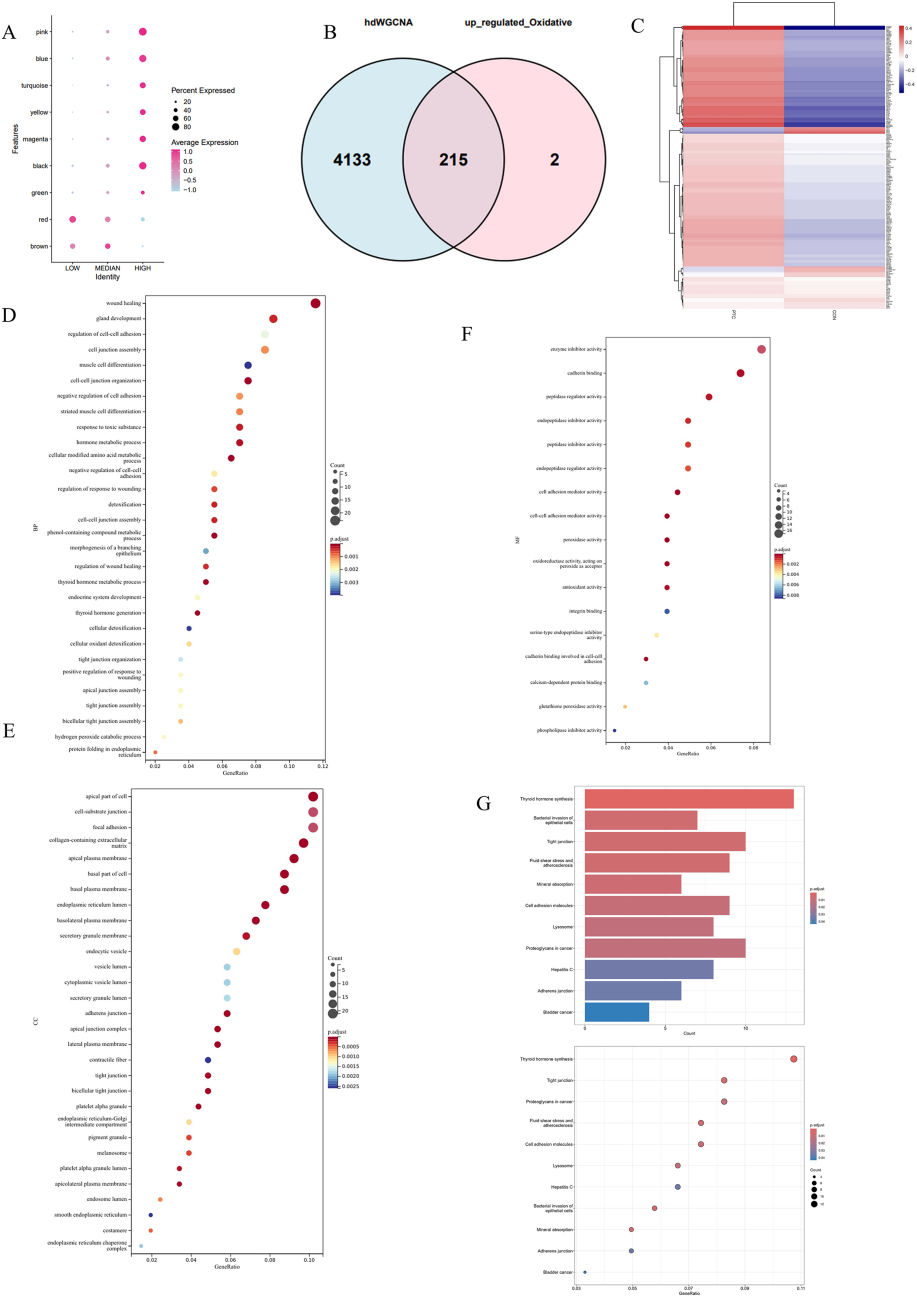
Functional enrichment analysis of overlapping genes identified by hdWGCNA and upregulated in the high OS group. **(A)** Dot plot showing the distribution and percentage of gene expression from various modules across cell populations classified by low, median, and high OS levels. **(B)** Venn diagram illustrating the overlap between genes identified by hdWGCNA (KME > 0.4) and genes upregulated in the high OS group. **(C)** Heatmap displaying the expression patterns of 215 overlapping genes. **(D-F)** GO enrichment analysis of the 215 overlapping genes, highlighting BP **(D)**, CC **(E)**, and MF **(F)** significantly associated with the overlapping genes. **(G)** KEGG pathway enrichment analysis showing significant pathways related to thyroid hormone synthesis, cancer biology, and immune evasion.

We explored the overlap genes identified by hdWGCNA and those upregulated in the high OS group, as well as the functional enrichment of these overlapping genes. A dot plot showed the distribution and percentage of gene expression from various modules across cell populations classified by low, median, and high OS levels. Key modules, such as pink, blue, turquoise, and black, were highly expressed in the OS-High group, while the brown module was predominantly expressed in the OS-Median group, and the red module was highly expressed in the OS-Low group. The highest average expression and percentage of expression were observed in the OS-High group, reinforcing the association between specific gene modules and OS activity in different cell populations within the PTC microenvironment (**Figure 5A**).

A Venn diagram illustrated the overlap between genes with KME > 0.4 identified by hdWGCNA and genes upregulated in the high OS group. Out of the 4,348 genes identified by hdWGCNA, 215 were upregulated in the high OS group, while only two genes were unique to the high OS dataset (**Figure 5B**).

To further explore the functional relevance of these overlapping genes, a heatmap was generated for the 215 genes. Hierarchical clustering revealed two distinct expression patterns: one group of genes was highly expressed in PTC and downregulated in CON. This pattern suggests that these genes may be drivers of OS-mediated responses in PTC (**Figure 5C**).

GO enrichment analysis was performed on the 215 overlapping genes, focusing on BP (**Figure 5D**), CC (**Figure 5E**), and MF (**Figure 5F**). In the BP category, key terms such as wound healing, gland development, cellular oxidant detoxification, and negative regulation of cell adhesion were significantly enriched. These processes are directly related to tissue repair and cancer progression, emphasizing the potential role of OS in promoting tumor growth and dysregulated tissue remodeling in PTC. For CC, enriched GO terms included collagen-containing extracellular matrix, apical plasma membrane, and cytoskeleton, all of which are involved in maintaining cellular structure and supporting tumor invasion and metastasis. This indicates that the overlapping genes may contribute to the physical changes in the tumor microenvironment that promote cancer cell proliferation and spread. MF analysis highlighted terms related to enzyme inhibitor activity, cadherin binding, and oxidoreductase activity. These functions are linked to cell adhesion, OS regulation, and enzyme activity, further suggesting that OS may drive molecular changes critical for PTC development and progression.

KEGG pathway enrichment analysis of the overlapping genes identified several important pathways, including thyroid hormone synthesis, bacterial invasion of epithelial cells, tight junction, and proteoglycans in cancer (**Figure 5G**). These pathways are crucial for thyroid-specific functions and general cancer biology, indicating that genes upregulated in response to OS may contribute to both PTC-specific and general cancer pathways, affecting tumor growth, immune evasion, and metastasis.

### Integrated multi-algorithm machine learning identifies *CCND1*, *SOX4*, and *TFF3* as robust core genes of the OS response

**Figure 6:**
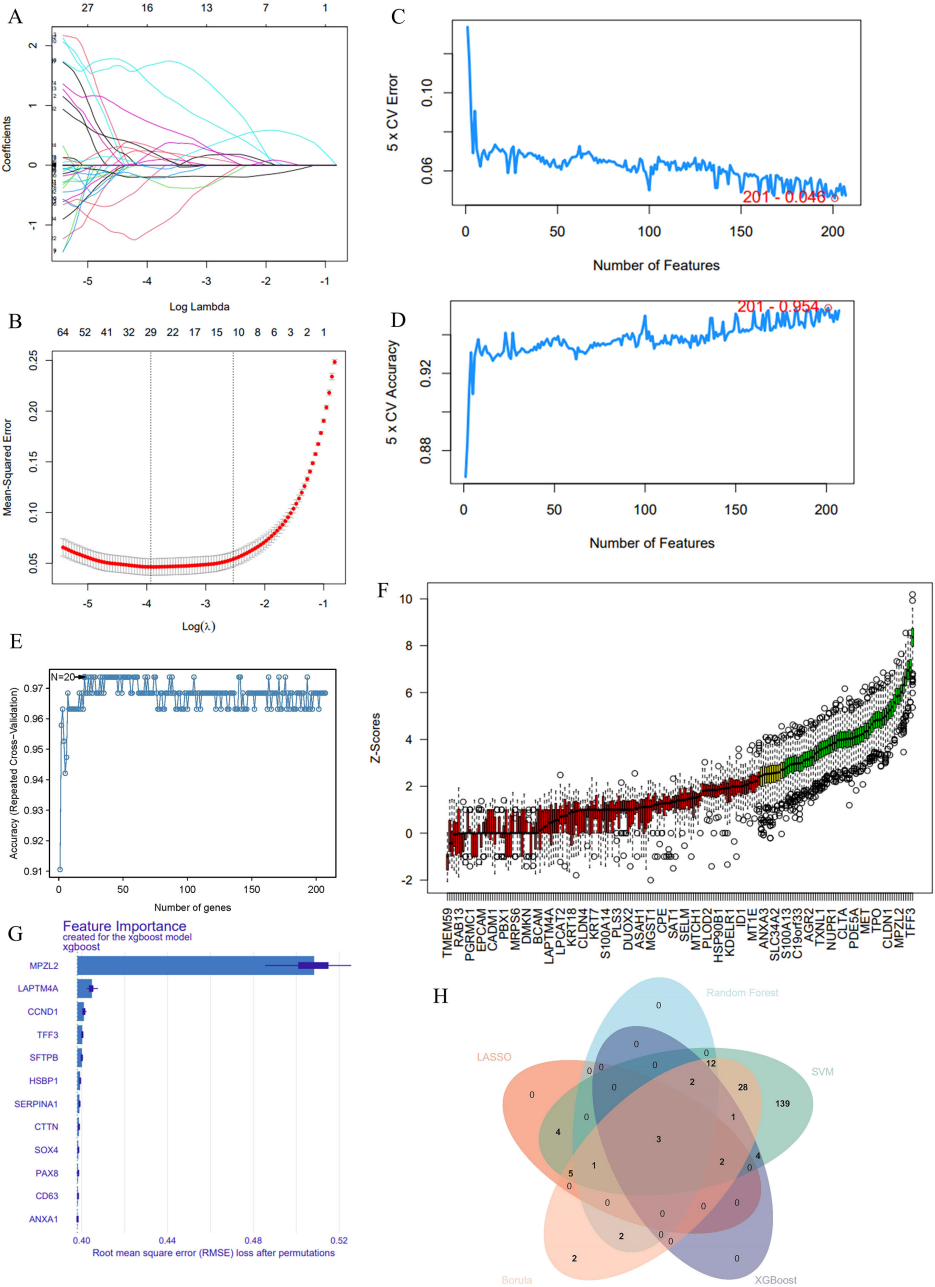
Multi-algorithm feature selection identifies key genes associated with OS-related PTC. **(A)** LASSO regression coefficient plot as a function of the regularization parameter (lambda), demonstrating how gene features are selected based on increasing lambda values. **(B)** Mean squared error plot from LASSO, indicating the optimal lambda value that minimizes error for selecting significant gene features. **(C)** Cross-validation error plot for SVM with RFE, showing the error rate across varying numbers of features. **(D)** Cross-validation accuracy plot for SVM with RFE, identifying the optimal number of features that maximize accuracy. **(E)** Random forest analysis of feature selection stability, indicating the number of features contributing to high predictive accuracy. **(F)** Boruta analysis ranking gene importance based on Z-scores, highlighting genes with the highest significance in OS-related PTC. **(G)** XGBoost feature importance plot showing the root mean square error (RMSE) loss after permutations, identifying genes such as MPL2G, LAPTM4A, and CCND1 as top contributors to PTC outcomes. **(H)** Venn diagram illustrating the overlap of selected genes across multiple machine learning algorithms, including LASSO, SVM, random forest, Boruta, and XGBoost. Three genes were consistently selected across all methods, emphasizing their potential as robust biomarkers for OS-related PTC.

We integrated data from GSE33630 and GSE60542, resulting in bulk RNA-seq data comprising 82 PTC and 75 CON samples. A LASSO regression model was used to identify significant gene features by shrinking the coefficients of less relevant genes to zero. The coefficient plot, as a function of the regularization parameter (lambda), demonstrated how the number of genes decreased with increasing lambda, ultimately selecting an optimal gene subset (**Figure 6A**). The mean squared error plot indicated that the minimum error was achieved at a specific λ value, leading to the selection of the most predictive gene features (**Figure 6B**).

We also applied a SVM with RFE. The cross-validation error and accuracy plots illustrated the model’s performance across varying numbers of features. The optimal number of genes was chosen based on minimizing cross-validation error and maximizing accuracy, ensuring a robust and efficient feature set for predicting PTC outcomes (**Figures 6C, D**). Random forest analysis was employed to evaluate the stability and predictive accuracy of the selected gene set, identifying 20 representative genes (**Figure 6E**). Boruta analysis, a feature selection method based on feature importance ranking, identified key genes with high Z-scores, highlighting their potential roles in PTC and their relevance to OS (**Figure 6F**). XGBoost, another machine learning algorithm, ranked the importance of genes based on their contribution to model prediction. Genes such as *MPL2G*, *LAPTM4A*, and *CCND1* exhibited the highest feature importance scores, indicating their crucial roles in the OS environment of PTC (**Figure 6G**).

Finally, a Venn diagram was generated to visualize the overlap of genes identified by multiple machines learning algorithms, including LASSO, SVM, random forest, Boruta, and XGBoost. This integrated approach allowed us to identify three cross-validated genes consistently emphasized across all methods, underscoring their potential as robust biomarkers of OS-related PTC (**Figure 6H**). This multi-algorithm approach ensures comprehensive and reliable gene selection for further study within the context of PTC pathogenesis.

### *CCND1* and *SOX4* are upregulated whereas *TFF3* is downregulated in PTC and correlates with clinical features

**Figure 7:**
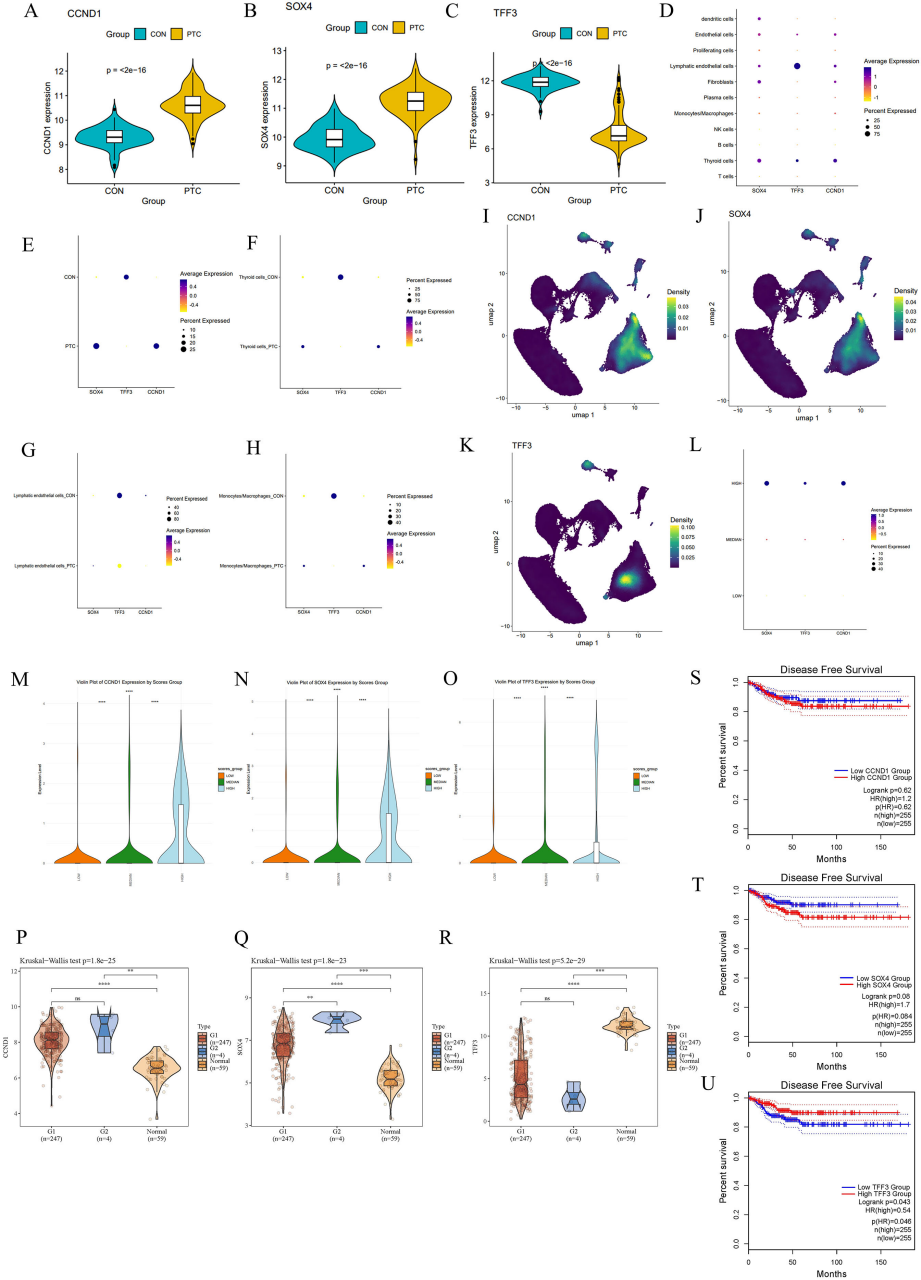
Validation of CCND1, SOX4, and TFF3 expression and their association with oxidative stress and clinical outcomes in PTC. **(A-C)** Violin plots depicting the expression levels of CCND1, SOX4, and TFF3 between PTC and CON. **(D)** Dot plot showing the expression of CCND1, SOX4, and TFF3 across different cell types in PTC and CON. **(E-H)** Dot plots displaying the percentage of cells expressing CCND1, SOX4, and TFF3 in thyroid cells and other cells across PTC and CON. **(I-K)** Density plots illustrating the distribution of CCND1, SOX4, and TFF3-expressing cells in PTC and CON groups. **(L)** dot plots showing the expression of CCND1, SOX4, and TFF3 across different OS group. **(M-O)** Violin plots of CCND1, SOX4, and TFF3 expression in cells categorized by oxidative stress (OS) levels: high, median, and low. **(P-R)** Violin plots depicting the expression of CCND1, SOX4, and TFF3 across different tumor stages based on TCGA data. **(S-U)** Kaplan-Meier survival curves indicating disease-free survival associated with high and low expression of CCND1, SOX4, and TFF3.

We validated the expression patterns, cellular distribution, and clinical relevance of the three key genes—*CCND1*, *SOX4*, and *TFF3*—in PTC and control samples across different datasets. Violin plots of gene expression indicated that *CCND1* and *SOX4* were significantly upregulated in PTC samples compared to controls, while *TFF3* was significantly downregulated in PTC (all p < 0.001) (**Figures 7A–C**).

Dot plots depicted the expression of these genes across different cell types. In PTC, *CCND1* and *SOX4* were more highly expressed in thyroid cells, whereas *TFF3* showed reduced expression in the same cell types. This pattern aligns with their overall expression trends, suggesting that these genes are specifically dysregulated in thyroid cells within the PTC microenvironment (**Figure 7D**). Further cellular localization analysis showed that *CCND1* and *SOX4* were predominantly expressed in thyroid cells and immune-related cells such as macrophages, while TFF3 was mainly expressed in lymphatic endothelial cells in the control group (**Figures 7E–H**).

UMAP projections illustrated the spatial distribution of cells expressing *CCND1*, *SOX4*, and *TFF3*. High-density regions indicated clusters of cells with elevated *CCND1* and *SOX4* expression, especially in PTC. Cells expressing *TFF3* were sparsely distributed, primarily in a subset of thyroid cells and lymphatic endothelial cells (**Figures 7I–K**). Dimplots further revealed differences in the expression and localization of these three genes between PTC and CON, with *TFF3* showing higher expression in controls, while *CCND1* and *SOX4* were highly expressed in PTC, particularly in thyroid cells (Supplementary **Figure 1C**).

All three genes exhibited the highest expression levels in the OS-High group, followed by the Median group, with the lowest expression observed in the Low group (**Figure 7L**). The distribution of gene expression based on OS score grouping showed that *CCND1* and *SOX4* had the highest expression in the high OS group. Although *TFF3* expression was also higher in the high OS group, the fold change was smaller. These findings support the association between these genes and the OS landscape in PTC, suggesting that *CCND1* and *SOX4* are involved in OS-driven tumorigenesis, while *TFF3* may act as a tumor suppressor in response to OS (**Figures 7M–O**).

We analyzed the expression levels of *CCND1*, *SOX4*, and *TFF3* across different tumor stages using TCGA data. Significant differences were observed between normal controls and tumors, with *CCND1* and *SOX4* showing higher expression in PTC, while *TFF3* was more highly expressed in normal samples. These results were consistent with our earlier conclusions. *SOX4* exhibited increasing expression trends in more advanced tumor stages, suggesting that *SOX4* may play roles in tumor progression and metastasis (**Figures 7P–R**). Survival analysis further emphasized the clinical relevance of these genes. Higher expression of *CCND1* and *SOX4* showed a trend toward poorer prognosis, although the difference was not statistically significant. In contrast, higher *TFF3* expression was correlated with better prognosis (*p* < 0.05) (**Figures 7S–U**).

### *CCND1* maintains a dedifferentiated stem-like phenotype and remodels the immune microenvironment via the PROS1-AXL signaling axis

**Figure 8:**
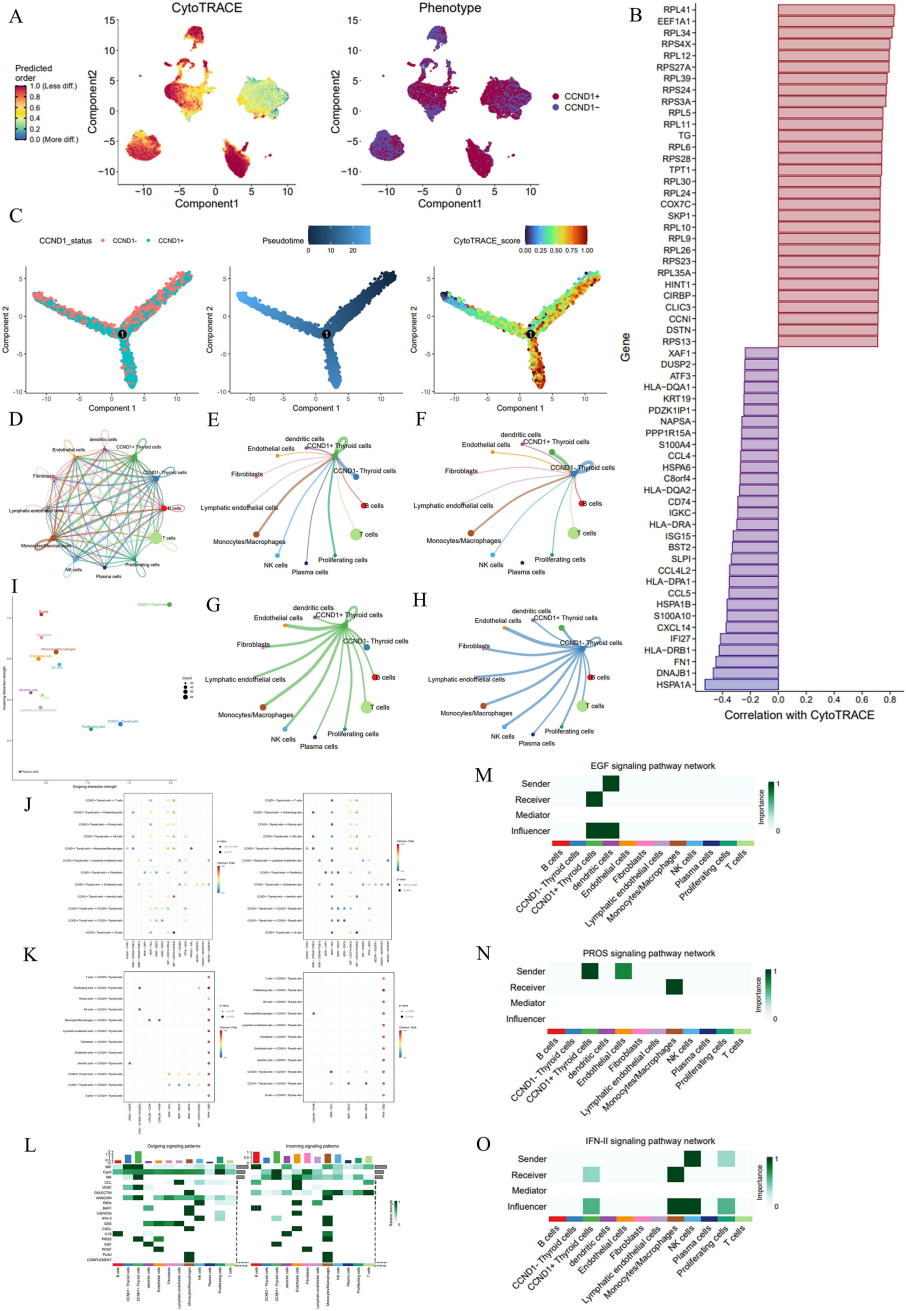
Analysis of CCND1+/- thyroid cell differentiation, gene expression, and signaling communication in PTC. **(A)** CytoTRACE and phenotypic maps showing differentiation states across PTC cells. Cells with high CytoTRACE scores are predicted to be less differentiated. **(B)** Bar plot depicting genes correlated with CytoTRACE scores. **(C)** Pseudotime plots highlighting thyroid cells at distinct stages of differentiation. **(D)** Network plot visualizing cellchat between different cell types in the PTC microenvironment using CellChat analysis. **(E-H)** Cell communication analysis showing the interactions between CCND1+/- thyroid cells and other cell types, including signal-sending and receiving dynamics. **(I)** Quantification of overall communication strength between various cell types. **(J)** Dot plot showing the receptor-ligand pathway for CCND1+/- thyroid cells as the source. **(K)** Dot plot showing the receptor-ligand pathway for CCND1+/- thyroid cells as the target. **(L)** Heatmap of signaling pathways in different cell types. **(M-O)** Analysis of specific pathways. EGF signaling network **(M)**, PROS signaling network **(N)**, IFN-II signaling network **(O)**.

We investigated the cellular differentiation trajectories and cell chat networks in PTC, with a focus on the role of *CCND1*. Using CytoTRACE and pseudotime analysis, we assessed the differentiation potential of PTC cells. The CytoTRACE map revealed a gradient of differentiation states among PTC cells, where a higher predicted order corresponded to a less differentiated state (**Figure 8A**). Notably, *CCND1*+ Thyroid cells were enriched in areas associated with lower differentiation states, suggesting that *CCND1* expression is linked to a stem-like, undifferentiated phenotype. Correlation analysis between gene expression and CytoTRACE scores identified key genes that positively or negatively correlated with cell differentiation. Genes like *RPL41* and *EEF1A1* exhibited strong positive correlations, associating them with less differentiated states, while genes such as *HSPA1A* and *DNAJB1* showed negative correlations, linking them to more differentiated cells. These differential expression patterns highlighted potential drivers of differentiation in the PTC microenvironment (**Figure 8B**).

Pseudotime analysis provided further insights into the dynamics of cell differentiation. In the pseudotime plot, *CCND1*+ Thyroid cells were primarily located at the root of the trajectory, indicating they represent early stages of differentiation (**Figure 8C**). As cells progressed along the pseudotime trajectory, *CCND1* expression significantly decreased, further supporting the association of *CCND1* with early, less differentiated cell states.

Cell chat analysis using the CellChat package revealed complex signaling interactions between various cell types in the PTC microenvironment (**Figure 8D**). When focusing on *CCND1*+ thyroid cells, they were found to engage in extensive communication with other cell types, including endothelial cells, fibroblasts, and immune cells. *CCND1*+ thyroid cells acted as both signal senders and receivers in multiple pathways, underscoring their central role in regulating the tumor microenvironment. Notably, plasma cells were identified as signal senders that specifically regulated *CCND1*+ thyroid cells, but not *CCND1*- Thyroid cells, suggesting a potential role for *CCND1* in immune regulation (**Figures 8E–H**).

Further analysis quantified the overall communication probability between various cell types, demonstrating that *CCND1*+ thyroid cells had strong abilities to both send and receive signals, while *CCND1*- thyroid cells were only primarily strong signal senders (**Figure 8I**). This indicates that *CCND1*+ thyroid cells actively shape the microenvironment through extensive signaling.

Pathway analysis revealed specific interactions involving *CCND1*+/- thyroid cells. A dot plot highlighted significantly increased signaling activity related to the PROS1-AXL pathway, where *CCND1*+ thyroid cells predominantly acted as signal senders. This suggests that *CCND1*+ thyroid cells may promote tumor growth and immune evasion through interactions with immune cells in the tumor microenvironment (**Figure 8J**). We also explored the signal transduction pathways in which *CCND1*+/- thyroid cells acted as receivers. Four pathways—AREG-EGFR, IFNG-(IFNGR1+IFNGR2), LGALS9-CD44, and MIF-(CD74+CD44)—were highly active when *CCND1*+ thyroid cells served as signal receivers. These findings suggest that *CCND1*+ thyroid cells interact with surrounding cells and factors through multiple signaling pathways, driving tumor growth, immune evasion, and invasion (**Figure 8K**).

A heatmap of the signaling network showed that *CCND1*+ thyroid cells exhibited enhanced communication both as senders and receivers. As signal senders, *CCND1*+ thyroid cells were significantly involved in the PROS and MK pathways, while as receivers, they were highly active in the EGF, IFN-II, and CypA pathways. This indicates that *CCND1*+ thyroid cells are more actively involved in intercellular communication than *CCND1*- thyroid cells, suggesting that *CCND1*+ thyroid cells not only regulate immune responses but also modulate their own proliferation, immune evasion, and adaptation to the microenvironment (**Figure 8L**).

We further examined the signaling network roles of different cell types in key pathways. In the EGF signaling network, dendritic cells were identified as major signal senders, while *CCND1*+ thyroid cells were the primary signal receivers (**Figure 8M**). In the PROS signaling network, *CCND1*+ thyroid cells were emphasized as key signal senders, underscoring their central role in modulating cellular behavior (**Figure 8N**). In the IFN-II signaling network, these cells acted as both receivers and influencers, mainly receiving regulatory signals from NK cells (**Figure 8O**).

### *SOX4* drives lineage dedifferentiation and orchestrates immunosuppressive crosstalk through the MDK signaling pathway

**Figure 9:**
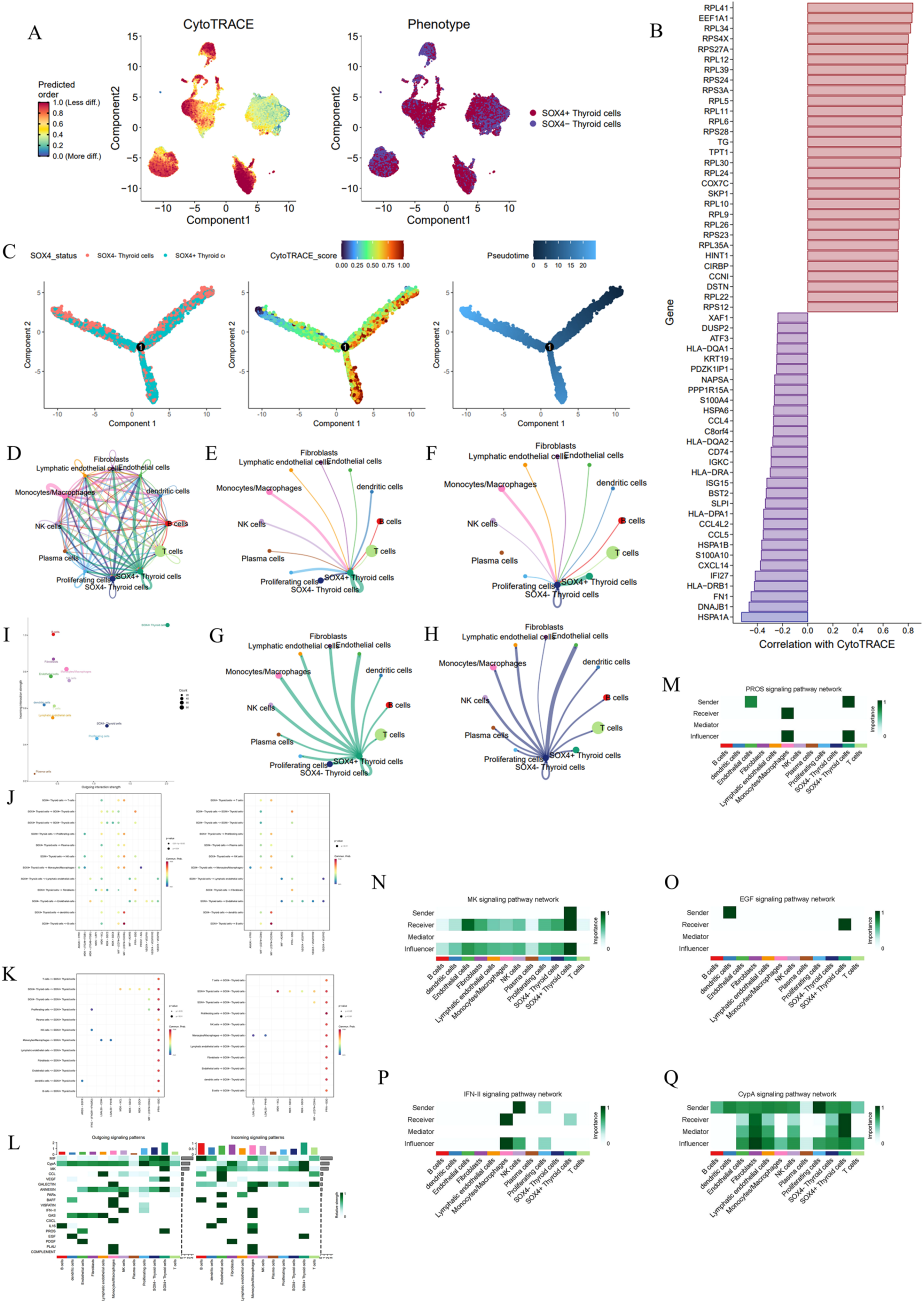
Analysis of SOX4+/- thyroid cell differentiation, gene expression, and signaling communication in PTC. **(A)** CytoTRACE map and phenotypic mapping showing differentiation states of SOX4+ and SOX4- thyroid cells. Cells with high CytoTRACE scores are predicted to be less differentiated. **(B)** Bar plot showing genes correlated with CytoTRACE scores, highlighting those positively and negatively associated with differentiation. **(C)** Pseudotime plots highlighting thyroid cells at distinct stages of differentiation. **(D)** Network plot visualizing cellchat between different cell types in the PTC microenvironment using CellChat analysis. **(E-H)** Detailed cellchat analysis showing signal-sending and receiving dynamics of SOX4+ and SOX4- thyroid cells. **(I)** Quantification of overall communication strength between various cell types. **(J)** Dot plot showing the receptor-ligand pathway for SOX4+/- thyroid cells as the source. **(K)** Dot plot showing the receptor-ligand pathway for SOX4+/- thyroid cells as the target. **(L)** Heatmap of signaling pathways in different cell types. **(M-Q)** Analysis of specific pathways. PROS1 signaling network **(M)**, MK signaling network **(N)**, EGF signaling network **(O)**, IFN-II signaling network **(P)**, and CypA signaling network **(Q)**.

The CytoTRACE map showed the differentiation potential and phenotypic characteristics of *SOX4*+ and *SOX4*- thyroid cells in the PTC microenvironment. The CytoTRACE map, which predicts cell differentiation potential, revealed a gradient where *SOX4*+ thyroid cells were mainly located in areas with lower differentiation degrees, indicating a more progenitor-like state. The phenotype map suggested that *SOX4*+ thyroid cells varied in differentiation levels and clustered in regions associated with early differentiation stages. This implies that *SOX4* may play a role in maintaining a less differentiated, stem-like cell phenotype in PTC (**Figure 9A**).

Correlation analysis between gene expression and CytoTRACE scores identified a list of genes positively or negatively correlated with cell differentiation. Genes such as *RPL41*, *EEF1A1*, and *RPS3A* were positively correlated with CytoTRACE scores, suggesting their association with less differentiated states and early cell processes or maintenance of progenitor cell status. Conversely, genes like *HSPA1A* and *DNAJB1* were negatively correlated, linking them to more differentiated cells. This pattern highlighted potential molecular drivers and differentiation markers within the PTC microenvironment (**Figure 9B**).

Pseudotime analysis illustrated the dynamics of differentiation in *SOX4*+ thyroid cells. In the pseudotime plot, *SOX4*+ thyroid cells (highlighted in the left panel) were positioned at the root of the trajectory, indicating an early differentiation state. The middle and right panels showed the pseudotime progression and CytoTRACE scores, further supporting the association of *SOX4* expression with an undifferentiated cell phenotype. These findings suggest that *SOX4*+ cells represent an early, stem-like state in the PTC differentiation hierarchy (**Figure 9C**).

A network map visualized the global cell chat patterns in the PTC microenvironment. *SOX4*+ thyroid cells displayed extensive interactions with various cell types, including fibroblasts, endothelial cells, and immune cells. This wide communication network suggests that *SOX4*+ thyroid cells play a central role in coordinating intercellular signaling within the tumor microenvironment, potentially influencing tumor growth, immune regulation, and matrix remodeling (**Figure 9D**).

Detailed analysis of the communication between *SOX4*+ and *SOX4*- thyroid cells revealed differences in their reception of signals from other cell types. Notably, plasma cells were found to communicate with *SOX4*+ thyroid cells but not with *SOX4*- thyroid cells (**Figures 9E, F**). *SOX4*+ thyroid cells exhibited stronger signaling activity as signal senders compared to SOX4- thyroid cells, indicating that *SOX4* may play an important role in cell signaling transduction (**Figures 9G, H**).

Quantification of the signaling strength between various cell types demonstrated that *SOX4*+ thyroid cells had significantly higher signal sending and receiving intensities, reinforcing the view that they are key participants in the tumor microenvironment signaling network. In contrast, *SOX4*- thyroid cells had much lower signaling intensities, whether as signal senders or receivers, underscoring the importance of *SOX4* in signaling (**Figure 9I**).

Dot plots further analyzed the signaling pathways in which *SOX4*+/- thyroid cells actively participated. In the MDK pathway (including: MDK−(ITGA4+ITGB1), MDK−(ITGA6+ITGB1), MDK−LRP1, MDK−NCL, MDK−SDC2, MDK−SDC4), *SOX4*+ thyroid cells acted as signal senders to various cells, including thyroid cells, endothelial cells, monocytes/macrophages, and NK cells, whereas *SOX4*- thyroid cells had no such interactions. In the PROS1-AXL pathway, *SOX4*+ thyroid cells could influence monocytes/macrophages, a unique feature of these cells. As signal receivers, *SOX4*+ thyroid cells had stronger connections in the PPIA-BSG pathway compared to SOX4- cells, and they received higher-intensity signals in the IFNG-(IFNGR1+IFNGR2) pathway from immune cells. The identification of specific ligand-receptor pairs highlighted the mechanisms by which *SOX4*+ thyroid cells exert their influence, offering insights into potential therapeutic targets for blocking these tumor-promoting signaling pathways (**Figures 9J, K**).

A heatmap demonstrated that *SOX4*+ thyroid cells possessed signaling capabilities in the PROS and MK pathways, which were absent in *SOX4*- thyroid cells, regulating other cell types through signal sending. As signal receivers, *SOX4*+ thyroid cells received signals from the IFN-II and EGF pathways, and their signal reception in the CypA pathway was stronger than that of *SOX4*- thyroid cells. *SOX4*+ thyroid cells were described as key mediators and influencers in these networks, emphasizing SOX4’s multifaceted role in the PTC microenvironment, not only in cell differentiation but also in regulating tumor growth and immune responses (**Figure 9L**).

We further analyzed the role of *SOX4*+ thyroid cells in key pathways. In the PROS pathway, *SOX4*+ thyroid cells served as core signal senders and influencers, suggesting their potential role in extracellular matrix remodeling and metastatic potential (**Figure 9M**). In the MK-mediated signaling pathway, *SOX4*+ thyroid cells were highlighted as major signal senders, driving proliferation and angiogenesis signals in the microenvironment (**Figure 9N**). In the EGF-mediated signaling pathway, *SOX4*+ thyroid cells received signals from dendritic cells, indicating their involvement in immune-related tumor progression pathways (**Figure 9O**). Lastly, the IFN-II and CypA-mediated signaling pathways indicated that *SOX4*+ thyroid cells were important receivers in these pathways, playing roles in immune regulation and interaction with inflammatory pathways (**Figures 9P, Q**).

### Loss of *TFF3* characterizes a distinct dedifferentiated state and is associated with altered immune-evasive signaling shifts

**Figure 10:**
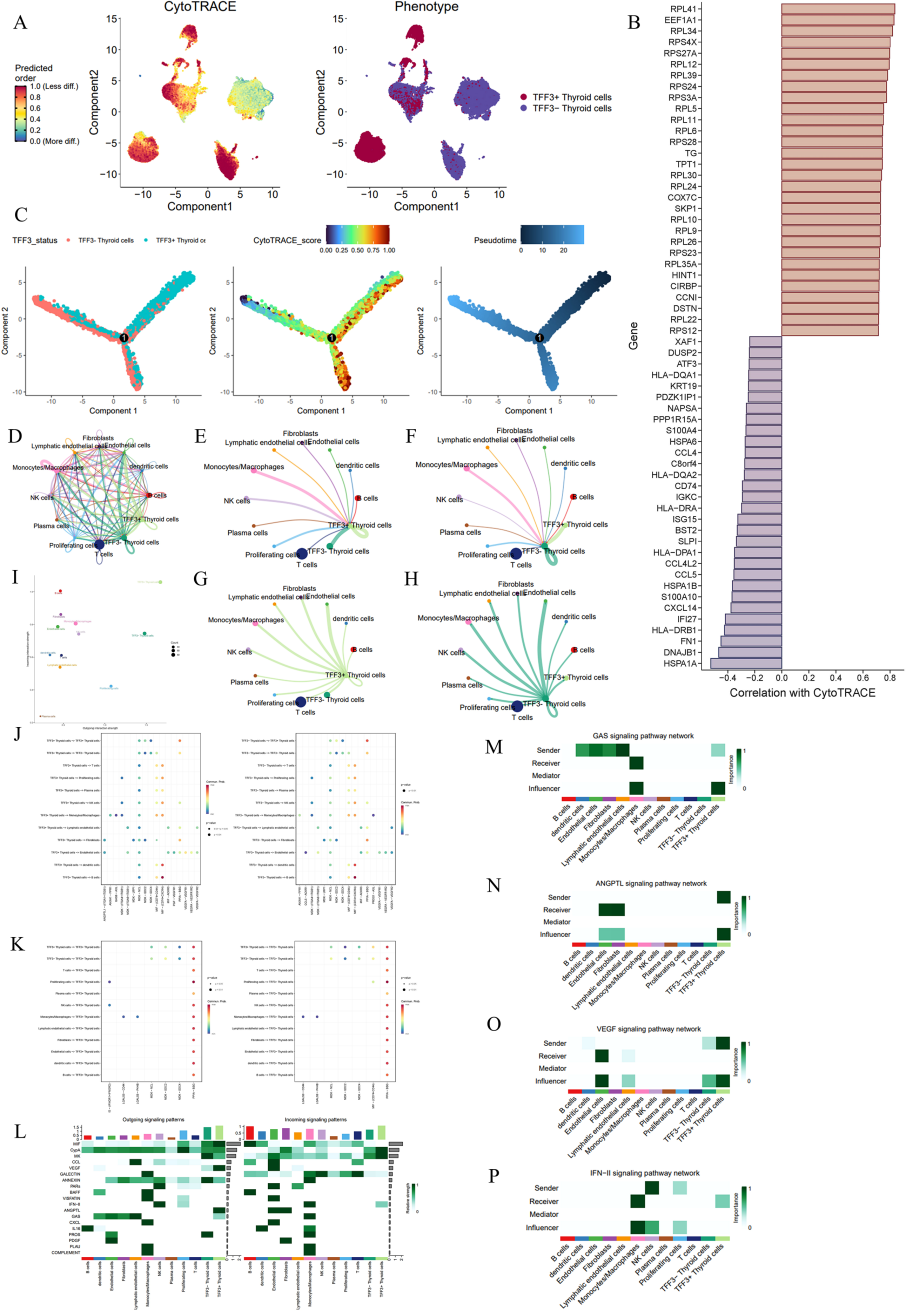
Analysis of TFF3+/- thyroid cell differentiation, gene expression, and signaling communication in PTC. **(A)** CytoTRACE map and phenotypic mapping showing differentiation states of TFF3+ and TFF3- thyroid cells. Cells with high CytoTRACE scores are predicted to be less differentiated. **(B)** Bar plot showing genes correlated with CytoTRACE scores, highlighting those positively and negatively associated with differentiation. **(C)** Pseudotime plots highlighting thyroid cells at distinct stages of differentiation. **(D)** Network plot visualizing cellchat between different cell types in the PTC microenvironment using CellChat analysis. **(E-H)** Detailed cellchat analysis showing signal-sending and receiving dynamics of TFF3+ and TFF3- thyroid cells. **(I)** Quantification of overall communication strength between various cell types. **(J)** Dot plot showing the receptor-ligand pathway for TFF3+/- thyroid cells as the source. **(K)** Dot plot showing the receptor-ligand pathway for TFF3+/- thyroid cells as the target. **(L)** Heatmap of signaling pathways in different cell types. **(M-P)** Analysis of specific pathways. GAS signaling network **(M)**, ANGPTL signaling network **(N)**, VEGF signaling network **(O)**, IFN-II signaling network **(P)**.

CytoTRACE and phenotype maps highlighted the differentiation status of *TFF3*+/- thyroid cells within the PTC microenvironment. The CytoTRACE map revealed a gradient of differentiation potential, with both *TFF3*+ and *TFF3*- thyroid cells occupying regions of both high and low differentiation, though they resided in different cell clusters. This suggests that both cell phenotypes are present across various differentiation stages, but their functions may differ (**Figure 10A**). Correlation analysis between gene expression and CytoTRACE scores identified genes positively or negatively correlated with cell differentiation. Genes like *RPL41* and *EEF1A1* showed a strong positive correlation with CytoTRACE scores, indicating their association with more differentiated cell states. Conversely, *HSPA1A* and *DNAJB1* exhibited a negative correlation, linking them to less differentiated, progenitor-like states (**Figure 10B**).

Pseudotime analysis further revealed the dynamics of differentiation in *TFF3*+ thyroid cells. In the left panel, *TFF3*+ thyroid cells were positioned in the pseudotime trajectory, suggesting their involvement earlier in cell development. The middle panel, displaying CytoTRACE scores, emphasized the association of *TFF3*+ thyroid cells with earlier developmental stages. The right panel indicated that early cell development may be primarily driven by *TFF3* (**Figure 10C**).

The cell chat network in the PTC microenvironment revealed extensive interactions between various cell types, but there was no significant difference in the interaction partners or intensity between *TFF3*+ and *TFF3*- thyroid cells. This may suggest that *TFF3* plays a limited role in regulating the microenvironment (**Figures 10D–H**). Quantification of communication intensity between different cell types showed that *TFF3*+ thyroid cells had the strongest interactions with other cell populations, while *TFF3*- thyroid cells exhibited slightly lower interaction intensity, indicating that *TFF3* may not be tightly involved in shaping the tumor microenvironment (**Figure 10I**).

A dot plot detailed the specific signaling pathways involving *TFF3*+ thyroid cells. The communication patterns between *TFF3*+ and *TFF3*- thyroid cells and other cell types varied, encompassing multiple signaling pathways. *TFF3*+ thyroid cells showed enhanced communication through pathways such as ANGPTL1-(ITGA1+ITGB1) with fibroblasts and endothelial cells, PGF-VEGFR1 with endothelial cells, and PROS1–AXL with fibroblasts. In contrast, *TFF3*- thyroid cells exhibited increased communication through the CCL5-ACKR1 pathway with endothelial cells and the PROS1-AXL pathway with monocytes/macrophages (**Figure 10J**). As signal receivers, *TFF3*+ thyroid cells were mainly characterized by receiving signals from proliferating cells and NK cells through the IFNG-(IFNGR1+IFNGR2) pathway, while *TFF3*- thyroid cells mainly received signals through the MIF-(CD74+CD44) pathway from both *TFF3*+/- thyroid cells and proliferating cells.

In the PPIA-BSG pathway, *TFF3*+ thyroid cells exhibited stronger signaling than *TFF3*- thyroid cells (**Figure 10K**). *TFF3*+ thyroid cells showed enhanced signal reception in multiple pathways, including CypA, MK, and IFN-II. Particularly in the IFN-II pathway, TFF3+ thyroid cells had significantly stronger signal reception compared to *TFF3*- thyroid cells, while *TFF3*- thyroid cells exhibited stronger signal reception in the MIF pathway. *TFF3*+ thyroid cells were more active in signal sending, particularly in the ANGPTL, GAS, and VEGF pathways, whereas *TFF3*- thyroid cells were more active in the PROS, MK, and ANNEXIN pathways, especially in the PROS pathway, which did not involve *TFF3*+ thyroid cells (**Figure 10L**). In the GAS signaling pathway, *TFF3*+ thyroid cells acted primarily as signal senders and influencers, whereas *TFF3*- thyroid cells had no notable role in the GAS pathway (**Figure 10M**). In the ANGPTL pathway, *TFF3*+ thyroid cells were the main signal senders and influencers, regulating endothelial cells and fibroblasts, highlighting their critical role in angiogenesis and cell-to-cell communication. *TFF3*- thyroid cells had a limited role in this pathway, suggesting a weaker function in this process (**Figure 10N**). *TFF3*+ thyroid cells acted as the primary signal senders in the VEGF pathway, indicating their potential involvement in angiogenesis (**Figure 10O**). In the IFN-II pathway, *TFF3*+ thyroid cells acted as signal receivers, suggesting that they are subject to regulation by other cells in the immune microenvironment. *TFF3*- thyroid cells played a lesser role in this pathway, implying their reduced involvement in immune responses (**Figure 10P**).

### *CCND1* acts as a central regulatory hub interacting with *SOX4* and *TP53* within the OS network

We used STRING to analyze the interaction network between *CCND1*, *SOX4* and *TFF3*. *CCND1* was located at the center of the network, showing complex interactions with multiple genes, including *TP53*, *SOX4*, and *TFF3*, suggesting *CCND1* plays a central regulatory role in this gene network (**Supplementary Figure 2A**). Correlation analysis revealed that *CCND1* was positively correlated with *SOX4* (R = 0.52, p < 0.05) and negatively correlated with *TFF3* (R = -0.2, p < 0.05). Additionally, *SOX4* was negatively correlated with *TFF3* (R = -0.4, p < 0.05). Furthermore, *CCND1* was positively correlated with *TP53* (R = 0.46, *p* < 0.05), as was *SOX4* (R = 0.5, *p* < 0.05). However, *TFF3* showed no significant correlation with *TP53* (**Supplementary Figures 2B–G**).

### OS functionally drives *CCND1* overexpression to promote cell proliferation and sustain ROS levels

**Figure 11:**
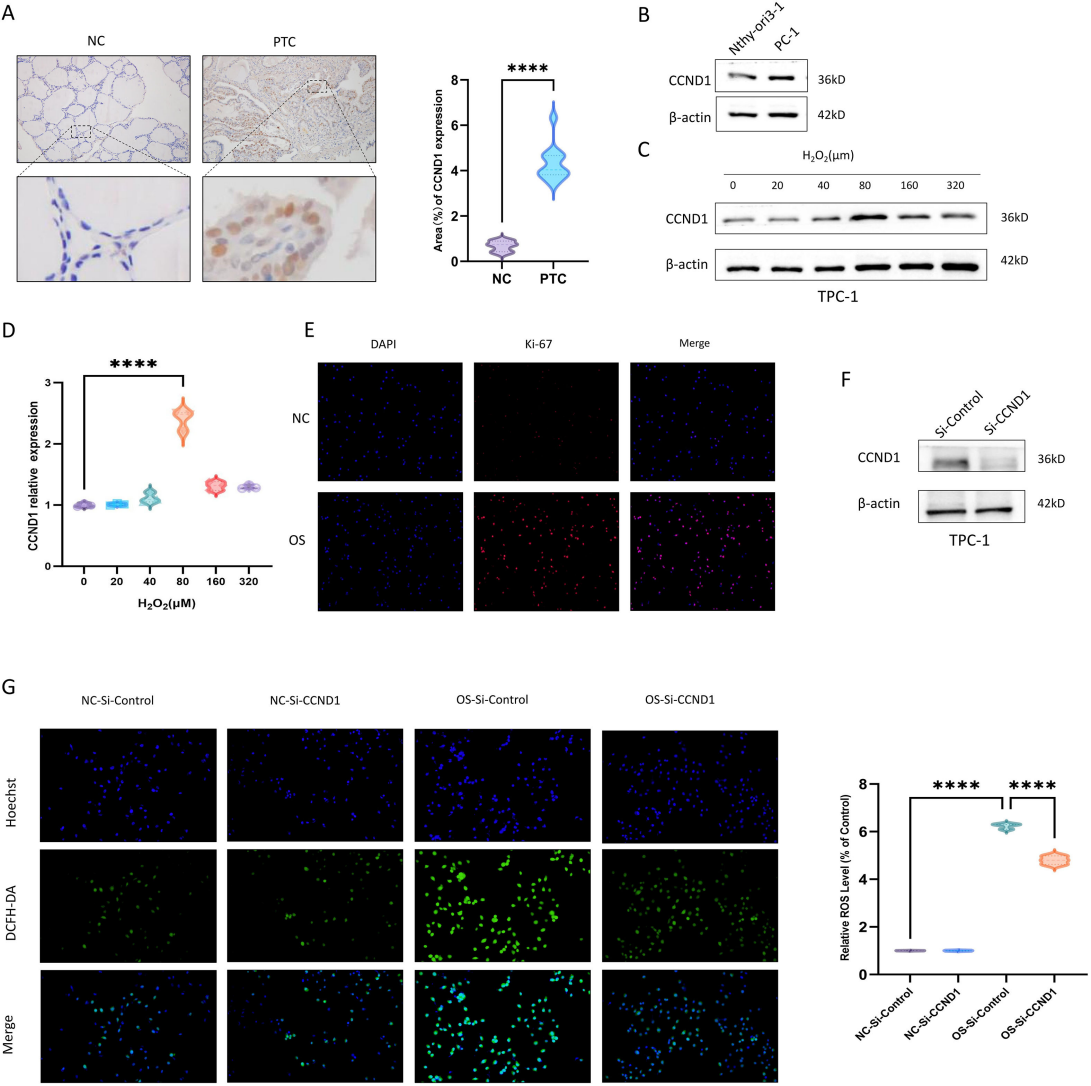
**(A)** Representative immunohistochemical staining images of CCND1 in papillary thyroid carcinoma and adjacent normal tissue sections, along with statistical significance analysis. **(B)** Representative Western blot images showing the relative expression levels of CCND1 in Nthy-ori3-1 and TPC-1cells. **(C)** Western blot analysis and **(D)** quantification of CCND1 expression in TPC-1 cells under treatment with increasing concentrations of H_2_O_2_. A significant increase in CCND1 expression was observed at 80 μM H_2_O_2_. **(E)** Proliferation capacity of TPC-1 cells under 80 μM H_2_O_2_-induced oxidative stress, as assessed by immunofluorescence staining: blue represents DAPI (nuclei) and red indicates Ki-67 (proliferation marker). **(F)** Western blot analysis of CCND1 expression in TPC-1 cells transfected with either scrambled non-targeting siRNA (Si-Control) or CCND1-targeting siRNA (Si-CCND1). **(G)** Measurement of intracellular ROS levels using the DCFH-DA probe (green) in TPC-1 cells transfected with Si-Control or Si-CCND1, under both basal and oxidative stress conditions. Group abbreviations: NC (Normal Condition, without H_2_O_2_ treatment); OS (Oxidative Stress, with 80 μM H_2_O_2_ treatment); Si-Control (Scrambled non-targeting siRNA). Nuclei were counterstained with Hoechst (blue). ****P < 0.0001.

CCND1 expression was significantly upregulated in clinical PTC specimens compared to normal thyroid tissues (**Figure 11A**). Consistent with this observation, CCND1 levels were markedly elevated in the PTC-derived cell lines TPC-1 relative to the normal thyroid follicular epithelial cell line Nthy-ori3-1 (**Figure 11B**). We hypothesized that this differential expression pattern may be associated with the high ROS environment characteristic of PTC tumors. To test this, we exposed TPC-1 cells to hydrogen peroxide (H_2_;O_2_;)-induced oxidative stress. Treatment with 80 μM H_2_;O_2_; for 1 hour resulted in a significant increase in CCND1 expression, accompanied by enhanced cellular proliferation (**Figures 11C–E**), supporting the notion that moderate oxidative stress can promote CCND1 expression and proliferation in PTC cells.

We next identified a specific small interfering RNA (siRNA) sequence that effectively knocked down CCND1 expression (**Figure 11F**). Notably, under the same oxidative stress conditions (80 μM H_2_;O_2_;, 1 hour), TPC-1 cells transfected with CCND1-targeting siRNA exhibited a significant reduction in intracellular ROS levels, as measured by DCFH-DA fluorescence intensity (1:3000, Beyotime, #S0034S), compared to cells transfected with a negative control siRNA (**Figure 11G**).

In summary, our integrated analysis reveals a comprehensive landscape where oxidative stress acts as a central driver in the PTC microenvironment, predominantly affecting thyroid cells and monocytes/macrophages. We identified a core gene signature comprising *CCND1*, *SOX4*, and *TFF3*, which are differentially regulated under high OS conditions and are closely linked to tumor progression, dedifferentiation, and immune evasion. Specifically, *CCND1* and *SOX4* were upregulated in high-OS PTC cells and associated with stem-like phenotypes and immunosuppressive signaling (e.g., PROS1-AXL, MDK), whereas *TFF3* was downregulated and linked to distinct angiogenic and immune interactions. Our *in vitro* validation further confirmed that OS upregulates CCND1, promoting cell proliferation and creating a feedback loop that sustains high ROS levels. Collectively, these findings highlight a complex interplay between oxidative stress, oncogenic gene regulation, and microenvironmental remodeling, providing a rationale for the mechanistic exploration and therapeutic targeting discussed below.

## Discussion

Our single-cell transcriptomic landscape reveals that the pathogenesis of PTC is driven by a profound reprogramming of the TME orchestrated by OS. Rather than acting in isolation, we propose that the upregulation of oncogenes *CCND1* and *SOX4*, coupled with the loss of the differentiation marker *TFF3*, creates a self-reinforcing network. This network not only promotes tumor cell proliferation but also actively remodels immune niches via paracrine signaling axes such as PROS1-AXL.

These findings are consistent with existing literature reporting the oncogenic roles of *CCND1* and *SOX4* in PTC. *CCND1*, a key regulator of the cell cycle, may drive the rapid proliferation of PTC cells through its overexpression, while the upregulation of *SOX4* is closely associated with increased tumor invasiveness. In contrast, the downregulation of *TFF3* in PTC suggests that it may inhibit tumor progression through specific mechanisms.

*CCND1* is crucial for the transition of the cell cycle from the G1 phase to the S phase. It forms a complex with cyclin-dependent kinases (CDK4 and CDK6), which phosphorylates the retinoblastoma protein (Rb), driving cell cycle progression. Overexpression of *CCND1* can lead to uncontrolled cell proliferation and may also contribute to increased tumor metastasis and drug resistance (34, 35). In PTC, *CCND1* overexpression promotes malignant transformation and progression by enhancing cell proliferation and evading apoptosis (36). Some researchers have proposed that *CCND1* splice variants could serve as diagnostic and prognostic biomarkers for PTC (37). Through CytoTRACE and pseudotime analysis, we found that *CCND1*+ thyroid cells were primarily enriched in less differentiated regions, further supporting the potential role of *CCND1* as a tumor stem cell marker. *In vitro* experiments further demonstrated that increased expression of CCND1 induced by oxidative stress was associated with enhanced proliferative activity in TPC-1 cells. Additionally, in other cancers, *CCND1* overexpression has been shown to promote cell proliferation, migration, and invasion. The oncogenic function of *CCND1* largely depends on the activation of signaling pathways such as MAPK and PI3K/AKT. These pathways not only enhance *CCND1* expression but also inhibit its ubiquitination and degradation, thereby prolonging its stability (34, 35, 38). In addition, we discovered that appropriate levels of OS further promote *CCND1* upregulation, creating a bidirectional feedback mechanism: on one hand, ROS stimulate *CCND1* expression, while on the other hand, *CCND1* overexpression increases cancer cells’ resistance to ROS, fueling the continued progression of cancer (39, 40).

In this study, we identified OS as a critical upstream driver of *CCND1* overexpression in PTC. Our functional validation confirmed that elevated OS levels directly promote CCND1 expression. Mechanistically, this upregulation is likely sustained by ROS-mediated activation of MAPK and PI3K/AKT pathways, which not only enhance *CCND1* transcription but also inhibit its ubiquitination and degradation, thereby prolonging its stability (34, 35, 38). While *CCND1* is canonically known for driving the G1-S transition via Rb phosphorylation, in the context of high OS, its upregulation likely represents a critical survival adaptation rather than a simple proliferative signal. Existing literature indicates that *CCND1* plays a pivotal non-canonical role in redox homeostasis: it prevents cancer cells from undergoing premature senescence by maintaining intracellular ROS at tolerable, sub-lethal levels (41). Depletion of CCND1 has been shown to trigger unregulated ROS accumulation and senescence via the p38-JNK pathway, independent of Rb status (41). Therefore, we propose a bidirectional adaptive loop in PTC: the oxidative tumor microenvironment induces *CCND1* amplification, which, in turn, acts as a stress buffer. This buffer function prevents the high ROS levels from reaching a lethal threshold that would trigger senescence, thereby enabling PTC cells to sustain proliferation despite substantial metabolic stress. This mechanism also explains the enrichment of *CCND1*+ cells in less differentiated trajectories, as evasion of senescence is a hallmark of stem-like plasticity.

This mechanistic insight exposes a dual therapeutic vulnerability. First, the direct dependence of PTC cells on *CCND1* for proliferation under high-OS conditions provides a strong rationale for repurposing CDK4/6 inhibitors (e.g., palbociclib or ribociclib). While these agents are established in breast cancer, our data suggests their efficacy in PTC might be stratified by the tumor’s oxidative status. Second, and perhaps more critically, our analysis reveals that *CCND1*+ cells hijack the PROS1-AXL axis to remodel the immune microenvironment. By engaging AXL on macrophages, these tumor cells likely enforce an M2-like immunosuppressive polarization (42, 43). Since Cabozantinib is already FDA-approved for radioiodine-refractory PTC, our findings provide a novel mechanistic basis to explain its clinical efficacy: immune reprogramming via PROS1-AXL blockade, and we advocate for its earlier use in high-risk, CCND1-high subgroups.

Beyond its regulation of the cell cycle and AXL signaling, our study reveals that *CCND1* overexpression actively coordinates a pro-tumorigenic secretome. The concomitant activation of AREG-EGFR and MIF-CD74 pathways suggests that *CCND1* functions as a central coordinator coupling intrinsic proliferation with environmental adaptation. Mechanistically, the AREG-EGFR axis establishes an autocrine loop that sustains PI3K-AKT survival signaling, reducing dependence on external growth factors (44). Notably, we noticed that EGFR can further amplify this invasive signaling through heterotypic interactions with AXL, creating a cooperative signaling node (45). In parallel, *CCND1* shapes the immune landscape through paracrine signaling. Secreted MIF interacts with CD74 to reinforce the immunosuppressive barrier, potentially synergizing with IFNG signaling to induce immune checkpoints like PDL1 and IDO1 (46). These insights provide a rationale for multi-modal therapeutic interventions. The reliance on the EGFR-AXL network suggests the utility of inhibitors like Cabozantinib, potentially in combination with CDK4/6 inhibitors (47). Furthermore, targeting the MIF-CD74 axis (e.g., via ibudilast) could dismantle the immunosuppressive niche (48). Collectively, these findings support a model where oxidative stress promotes *CCND1* overexpression, which in turn amplifies this autocrine and paracrine secretome to drive PTC progression.

Consistent with its established function in biology, our analysis identifies *SOX4* as a critical master regulator of lineage plasticity and dedifferentiation in PTC (49, 50). The preferential localization of *SOX4*-positive cells along less differentiated trajectories indicates that this transcription factor functions to arrest thyroid maturation, maintaining a stem-like state that fuels tumor self-renewal and heterogeneity. This dedifferentiated phenotype is likely sustained by the oxidative tumor microenvironment, where ROS-mediated signaling acts as a persistent stimulus for *SOX4* amplification via pathways such as PI3K, Wnt, and TGFβ (51). By promoting epithelial-mesenchymal transition (EMT) and maintaining cancer stem cell (CSC) properties, *SOX4* not only drives local invasiveness but also confers an intrinsic resistance to therapy, positioning it as a key determinant of tumor progression and recurrence (51–56).

Beyond regulating intrinsic cellular plasticity, *SOX4* actively remodels the tumor microenvironment through a multifaceted secretome, most notably via the Midkine (MDK) signaling axis. Our study reveals that *SOX4*-driven MDK secretion orchestrates a dual-pronged support system: it promotes angiogenesis by interacting with endothelial cells and, crucially, induces M2 polarization in macrophages to establish an immunosuppressive niche (57, 58). This intercellular crosstalk enables PTC cells to evade immune surveillance while securing nutrient supply. Furthermore, *SOX4* equips tumor cells with the metabolic flexibility required to thrive under high oxidative stress. Mechanistically, *SOX4* has been reported to upregulate HDAC1, which suppresses the thioredoxin-binding protein-2 (TBP-2), thereby enhancing antioxidant capacity (59, 60). Coupled with the regulation of Bcl-2 family proteins (61), this creates a pathological feedback loop where oxidative stress induces *SOX4*—potentially via EGFR-mediated MAPK/PI3K activation—which in turn fortifies the cell’s antioxidant defenses and anti-apoptotic threshold.

The identification of the *SOX4*-MDK axis as a central hub for immune evasion and dedifferentiation highlights its potential as a therapeutic target. Since *SOX4* acts as a transcription factor and is notoriously difficult to target directly with small molecules, interfering with its downstream effector mechanisms offers a more viable strategy. Specifically, targeting the MDK signaling pathway or its receptors could disrupt the pro-tumorigenic crosstalk between PTC cells and the immune microenvironment. Additionally, given the reliance of *SOX4*-high cells on HDAC1-mediated antioxidant mechanisms, the use of HDAC inhibitors might synthetically lethally target these dedifferentiated subpopulations, rendering them susceptible to oxidative cytotoxicity and improving the efficacy of conventional therapies (59).

TFF3 (Trefoil Factor 3) is traditionally recognized for its mucosal protective role, but it exhibits tumor-suppressing and tumor-promoting functions in retinoblastoma and cervical cancer, respectively (62, 63). Our study establishes that *TFF3* is significantly downregulated in PTC compared to normal tissues. Previous studies have mentioned that *TFF3* is a molecule essential for the differentiation of thyroid cells (64). Rather than functioning strictly as a classical tumor suppressor, we propose that the loss of TFF3 serves as a critical indicator of thyroid dedifferentiation and compromised follicular integrity. This downregulation aligns with the loss of differentiation phenotype often observed in aggressive PTC subtypes, suggesting that TFF3 levels could complement existing markers (like Tg and TPO) to stratify patient risk, particularly in identifying tumors prone to dedifferentiation.

Our single-cell analysis reveals a clinically relevant functional dichotomy linked to *TFF3* expression. While residual *TFF3*+ cells maintained angiogenic signaling (ANGPTL1-ITGA1), the predominant *TFF3*- subpopulation exhibited a distinct immune-evasive profile characterized by the upregulation of the CCL5-ACKR1 and PROS1-AXL axes. This functional shift opens specific avenues for precision therapy. Given that restoring differentiation markers like *TFF3* is therapeutically challenging, targeting the downstream vulnerabilities acquired upon its loss offers a pragmatic alternative. Specifically, the enrichment of the PROS1-AXL axis in *TFF3*- cells suggests that these tumors may be uniquely susceptible to AXL-targeting inhibitors (e.g., cabozantinib or bemcentinib) (47, 65). Mechanistically, AXL signaling is a well-established driver of intrinsic resistance to MAPK inhibitors and plays a pivotal role in maintaining an immunosuppressive macrophage niche (66, 67). Therefore, inhibiting this axis could effectively disrupt tumor-macrophage crosstalk, reversing the immune-suppressive microenvironment observed in *TFF3*-low PTC.

Furthermore, the concomitant upregulation of pro-inflammatory mediators (CCL5, IL-6/JAK/STAT) in *TFF3*- cells highlights a broader dependency on inflammatory signaling for survival. Clinically, this implies that *TFF3* status could serve as a stratification biomarker to identify patients who are candidates for immune-modulating therapies. We propose that patients with a “Low-*TFF3*/High-*PROS1*” signature might benefit from combinatorial regimens targeting these specific immune checkpoints, rather than standard monotherapies. To validate this translational potential, future retrospective studies should assess *TFF3* expression in PTC cohorts treated with AXL-targeting multikinase inhibitors. We hypothesize that low *TFF3* levels will correlate with superior clinical responses to such regimens, validating its utility as a predictive biomarker for targeted intervention.

Moreover, research by Kate et al. suggested that *TFF3* deficiency affects the expression of antioxidant proteins in the liver (68). Mouse studies also demonstrated that *TFF3* deficiency weakens vascular dilation, further supporting its potential role in regulating vascular function and OS (69). Although we observed a correlation between low TFF3 expression and high OS levels as well as tumor invasiveness, further functional validation is still needed to determine the causal relationship. It remains to be elucidated whether restoring TFF3 expression could rescue the phenotype or whether its loss is an irreversible consequence of malignant transformation.

In conclusion, our study delineates a complex regulatory network where oxidative stress drives the malignant evolution of PTC through the distinct modulation of *CCND1*, *SOX4*, and *TFF3*. We propose that *CCND1* and *SOX4* function as adaptive survival factors: their OS-induced upregulation not only fuels proliferation and stemness but also actively orchestrates an immunosuppressive microenvironment via the AREG-EGFR, MIF, and MDK signaling axes. Conversely, the significant downregulation of *TFF3* serves as a critical indicator of lineage dedifferentiation rather than merely a loss of tumor suppression. This loss of thyroid identity correlates with a shift toward immune-evasive communication patterns, such as the activation of CCL5-ACKR1 signaling, further compounding the hostile tumor niche. Collectively, these findings underscore oxidative stress as a central architect of PTC heterogeneity. Future therapeutic strategies should therefore focus on targeting these specific stress-adaptation mechanisms—such as the autocrine loops and secretome interactions identified herein—to dismantle the survival networks of aggressive PTC.

## Limitation

Despite this study revealing the important connections between OS and key genes in PTC, several limitations need to be considered. The heterogeneity of single-cell sequencing data may introduce some inaccuracies in cell type annotation and gene expression results. Therefore, future research should further explore the exact mechanisms of these genes in PTC through functional experiments and validation in clinical samples.

In addition, this study only validated CCND1 *in vitro* and lacked *in vivo* validation in experimental animals. Future research directions should include functional validation of *CCND1*, *SOX4*, and *TFF3* in PTC, particularly regarding their impact on tumor cell behavior under OS conditions in animal models. Moreover, the potential of these genes in clinical diagnosis and treatment should be further evaluated. Targeting OS or these key genes could offer new strategies and therapeutic targets for the treatment of PTC.

## Conclusion

In this study, we demonstrated that *CCND1* and *SOX4* are highly expressed in PTC, likely promoting tumor growth, invasion, and immune evasion by regulating OS and key signaling pathways such as PI3K/AKT and MAPK. In contrast, TFF3 is significantly downregulated in PTC, suggesting that it may exert tumor-suppressive effects by modulating immune responses and OS. A limitation of our current study is the absence of further validation and exploration of the proposed mechanisms through more in-depth *in vitro* and *in vivo* experiments. Addressing this gap will constitute the primary focus of our subsequent research efforts. Overall, we have identified an oxidative stress-related gene set in PTC. The relationship between PTC and OS warrants more extensive investigation, as it may offer novel diagnostic and therapeutic strategies in the future.

## Data Availability

The original contributions presented in the study are included in the article/**Supplementary Material**. Further inquiries can be directed to the corresponding authors.

## Glossary

PTC: Papillary Thyroid Carcinoma
OS: Oxidative Stress
ROS: Reactive Oxygen Species
GEO: Gene Expression Omnibus
scRNA-seq: Single-Cell RNA Sequencing
CON: Control
SNN: Shared Nearest Neighbor
UMAP: Uniform Manifold Approximation and Projection
PCA: Principal Component Analysis
GO: Gene Ontology
KEGG: Kyoto Encyclopedia of Genes and Genomes
hdWGCNA: High-Dimensional Weighted Gene Co-Expression Network Analysis
LASSO: Least Absolute Shrinkage and Selection Operator
SVM: Support Vector Machine
RFE: Recursive Feature Elimination
DFS: Disease-Free Survival
PPI: Protein-Protein Interaction
TCGA: The Cancer Genome Atlas
TAM: Tumor-Associated Macrophage
MAPK: Mitogen-Activated Protein Kinase
PI3K: Phosphatidylinositol 3-Kinase
EGFR: Epidermal Growth Factor Receptor
IFN: Interferon
IFNGR: Interferon Gamma Receptor
PDL1: Programmed Death-Ligand 1
IDO1: Indoleamine 2,3-Dioxygenase 1
iNOS: Inducible Nitric Oxide Synthase
FAS: Fas Cell Surface Death Receptor
FASL: Fas Ligand
MIF: Macrophage Migration Inhibitory Factor
EMT: Epithelial-Mesenchymal Transition
CDK: Cyclin-Dependent Kinase
Rb: Retinoblastoma Protein
ERK: Extracellular Signal-Regulated Kinase
JNK: c-Jun N-terminal Kinase
AREG: Amphiregulin
LGALS9: Galectin-9
CD74: Cluster of Differentiation 74
CD44: Cluster of Differentiation 44
AXL: AXL Receptor Tyrosine Kinase
PROS1: Protein S
MK: Midkine
CypA: Cyclophilin A
NCL: Nucleolin
ITGA/ITGB: Integrin Alpha/Integrin Beta
LRP1: Low Density Lipoprotein Receptor-Related Protein 1
SDC2/SDC4: Syndecan 2/Syndecan 4
GAS: Growth Arrest-Specific Protein
ANNEXIN: Annexin Family.
