# Supplementary material for: Single-cell and machine learning integration reveals OS-driven CCND1 promotes an aggressive phenotype in papillary thyroid carcinoma
Source: Front Immunol. 2026 Jan 14;16:1722524. doi: 10.3389/fimmu.2025.1722524 (PMC12871538; doi:10.3389/fimmu.2025.1722524)
Supplement: Supplementary file 4 [file Table1.docx]

# *Supplementary materials*

# Single-cell and machine learning integration reveals OS-driven CCND1 promotes an aggressive phenotype in papillary thyroid carcinoma

**Jiaxi Wang ^1 †^, Qingyi Zhu ^2†^, Jingyi Bie ^3^, Yueyu Han ^1^, Hanqing Liu ^4^ *, Chuang Chen ^1^ ***

^1^ Department of Breast and Thyroid Surgery, Renmin Hospital of Wuhan University, Wuhan, People's Republic of China.

^2^ Department of Cardiovascular Surgery, Renmin Hospital of Wuhan University, Wuhan, People's Republic of China.

^3^College of Medicine, Yan'an University, Yan'an, People's Republic of China.

^4^ Department of Thyroid Surgery, the First Affiliated Hospital, School of Medicine, Zhejiang University, Hangzhou, People's Republic of China.

*** Correspondence:**Hanqing Liu
liuhqwhu@foxmail.com

Chuang Chen
chenc2469@whu.edu.cn

† These authors contributed equally to this work.

**Keywords**: Papillary thyroid carcinoma, Oxidative stress, Single-cell RNA sequencing, CCND1, SOX4, TFF3.


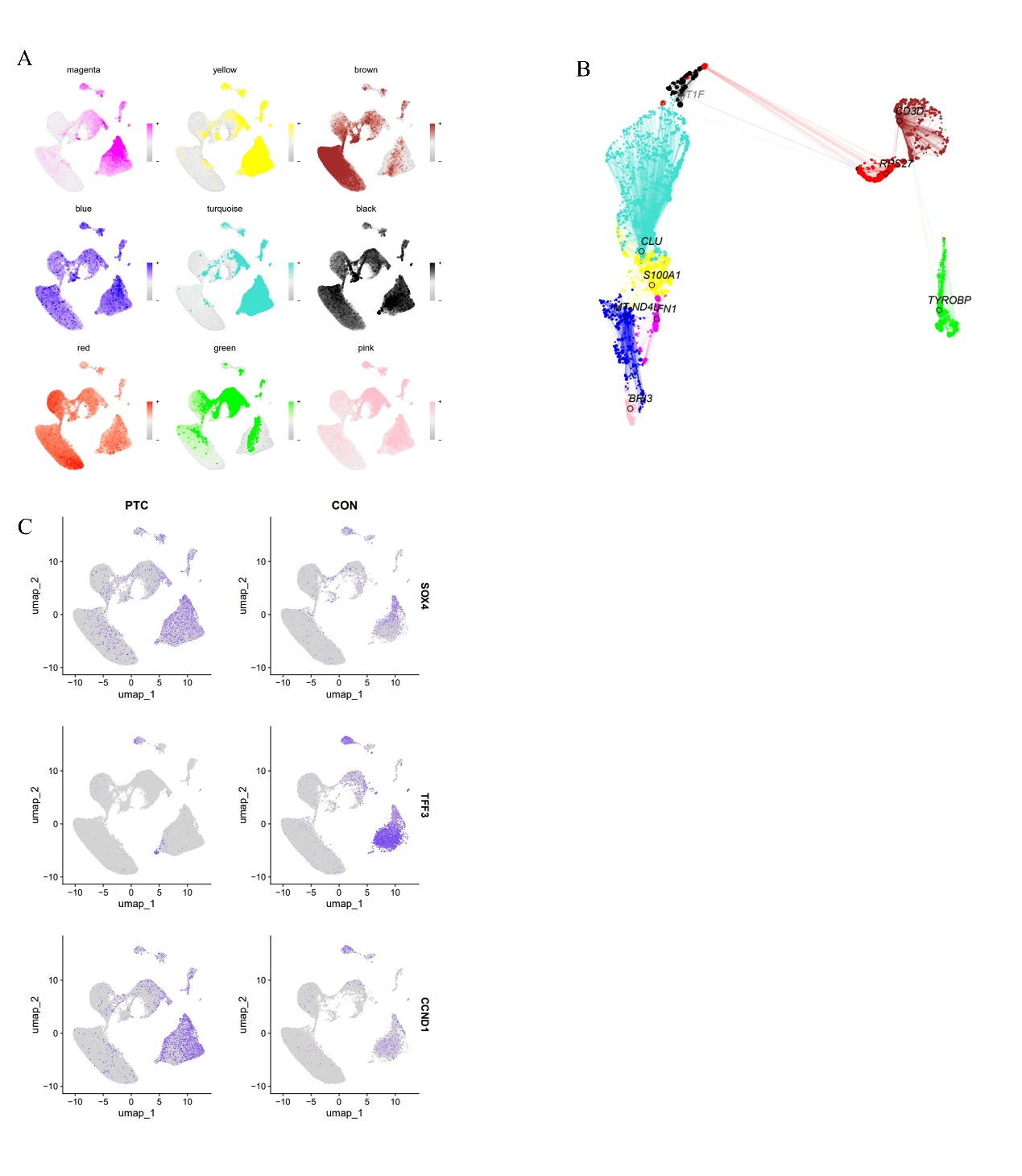
**Supplementary Figure 1**: Module and gene expression patterns in PTC and CON. (A) UMAP plots displaying the distribution of cells across different modules, with each module represented by a distinct color. (B) UMAP plot showing the relationships between representative genes from various modules, highlighting how distinct gene clusters relate to specific cell populations. (C) UMAP dimplots showing the expression of SOX4, TFF3, and CCND1 across PTC and CON. CCND1 and SOX4 are highly expressed in PTC samples, particularly in thyroid cells, while TFF3 is more highly expressed in CON.


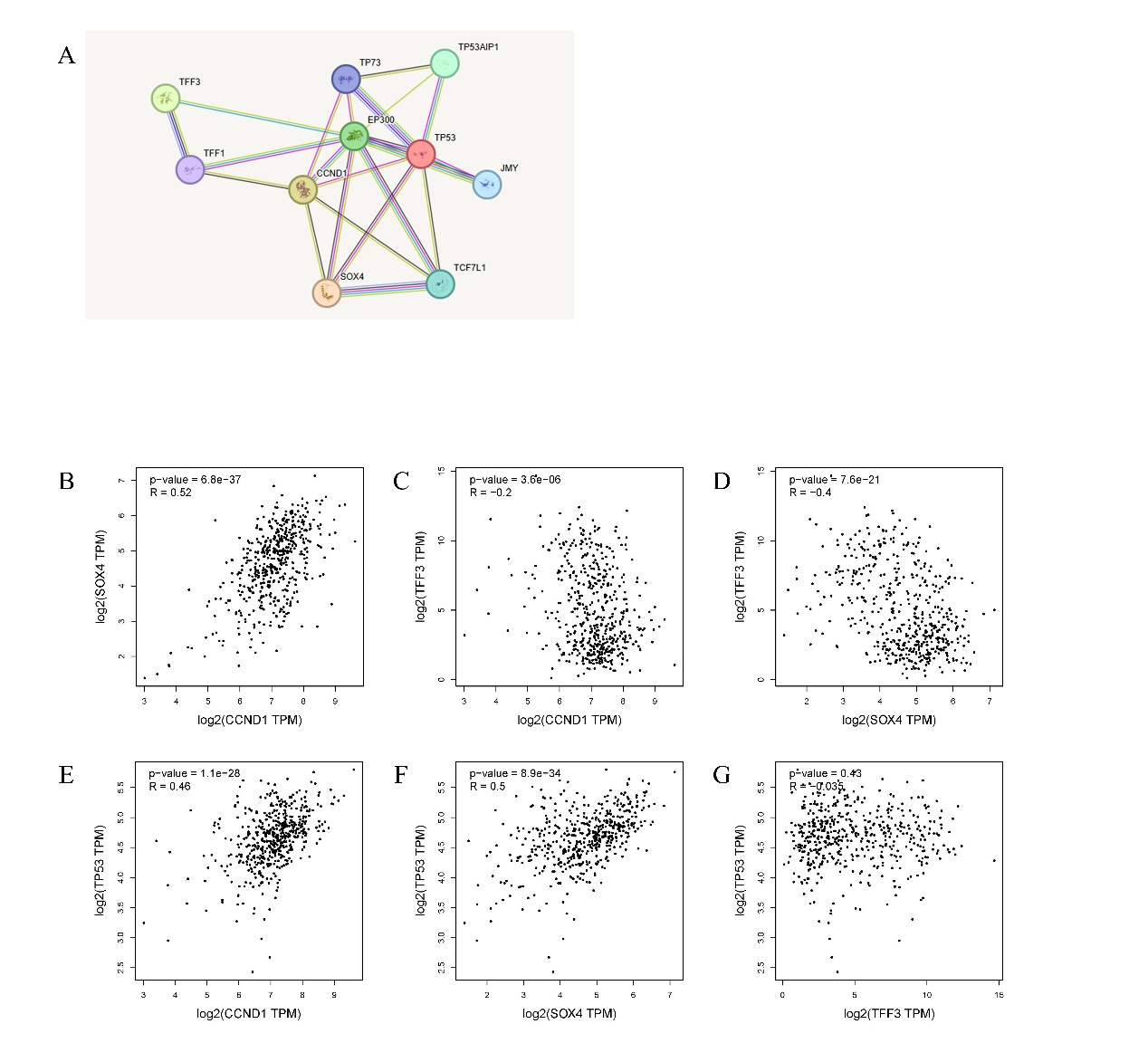


**Supplementary Figure 2**: Interaction network and correlation analysis of CCND1, SOX4, and TFF3 in PTC. (A) STRING interaction network showing the connections between CCND1, SOX4, TFF3, and other related genes, including TP53, TP53AP1, and JMY. (B-G) Correlation plots between key genes in the network. (B) Positive correlation between CCND1 and SOX4 (R = 0.52, p < 0.05). (C) Negative correlation between CCND1 and TFF3 (R = -0.2, p < 0.05). (D) Negative correlation between SOX4 and TFF3 (R = -0.4, p < 0.05). (E) Positive correlation between CCND1 and TP53 (R = 0.46, p < 0.05). (F) Positive correlation between SOX4 and TP53 (R = 0.5, p < 0.05). (G) No significant correlation between TFF3 and TP53 (R = 0.03, p > 0.05).

Supplement table 1: Differentially expressed genes between high OS group and low OS group.

*(This file is uploaded separately in CSV format)*

Supplement table 2: Module genes identified by hdWGCNA (kME>0.4).

*(This file is uploaded separately in CSV format)*

Supplement table 3: Overlapped genes identified by hdWGCNA and the upregulated DEGs in the high OS group.

*(This file is uploaded separately in CSV format)*
